# Occurrence and molecular characteristics of carbapenem-resistant Enterobacteriaceae recovered from wildlife: a systematic review and meta-analysis

**DOI:** 10.3389/fmicb.2026.1897815

**Published:** 2026-10-16

**Authors:** Ji-hwan Yim, Maryum Tanveer, Eurade Ntakiyisumba, Jin Hur, Gayeon Won

**Affiliations:** College of Veterinary Medicine and Bio-Safety Research Institute, Jeonbuk National University, Iksan, Republic of Korea

**Keywords:** antimicrobial resistance, carbapenem-resistant Enterobacteriaceae, meta-analysis, systematic review, wild animals

## Abstract

Carbapenem-resistant Enterobacteriaceae (CRE) represent a critical concern in the antimicrobial resistance (AMR) landscape due to their resistance to critical last-line antibiotics and their capacity for horizontal gene transfer. Although CRE are well described in clinical and livestock settings, evidence from wildlife remains scattered, limiting understanding of their ecological distribution outside managed systems. We conducted a systematic review and meta-analysis following PRISMA guidelines, screening 6,988 records published between January 2000 and July 2025 across three databases (PubMed, Scopus, and Web of Science), and 19 studies met the inclusion criteria. Using a random-effects model, the pooled CRE-positive proportion among recovered Enterobacteriaceae isolates tested for carbapenem resistance was estimated at 74.49% (95% CI: 49.75%–94.04%), with substantial between-study heterogeneity (*I*^2^ = 96%). However, this isolate-level estimate should not be interpreted as the prevalence of CRE carriage among wild animals. Species-level stratification revealed CRE-positive proportions of 76.51% for *Escherichia coli*, 72.41% for *Klebsiella* spp., and 86.46% for other Enterobacteriaceae genera, including *Citrobacter* and *Enterobacter* spp.; however, these subgroup estimates should be interpreted cautiously, as confidence intervals were wide and heterogeneity remained substantial across several analyses. The highest descriptive estimate was observed in water-associated environments (84.49%), while differences among sampling environments were not statistically significant. Although pooled estimates varied descriptively across host taxonomic order, carbapenem agent, continent, and phenotypic susceptibility testing method, only sample type showed a statistically significant subgroup difference (*p* < 0.01), while univariate meta-regression explained limited between-study variability overall. Clinically important carbapenemase-encoding genes, including *bla*NDM, *bla*KPC, *bla*OXA-48-like, *bla*VIM, and *bla*IMP, and diverse sequence types were reported across continents. Given the highly restricted veterinary use of carbapenems, these findings are consistent with potential exposure to resistant bacteria or resistance determinants originating from anthropogenically influenced environments; however, transmission direction cannot be established from the available evidence. Overall, wildlife may serve as useful sentinels of environmental CRE contamination, while longitudinal surveillance and genomic transmission studies are needed to clarify their role in CRE persistence and dissemination.

## 1 Introduction

Antimicrobial resistance (AMR) has been a serious global health concern in human and veterinary medicine since the early days of antibiotic treatment (McEwen and Collignon, 2018). Observations from the 20th century already documented reduced treatment efficacy and recurrent infections following antimicrobial therapy, and the problem has worsened considerably, with antimicrobial use and misuse representing major selective pressures contributing to the emergence and spread of resistance (French, 2010). In 2021 alone, an estimated 4.71 million deaths were associated with bacterial AMR (Naghavi et al., 2024). Although most attention has focused on human and livestock populations, antimicrobial-resistant bacteria are increasingly being reported in wildlife as well (Li et al., 2024). Among resistant bacterial threats, carbapenem resistance has become especially alarming. Carbapenems, which are often considered the last-resort broad-spectrum β-lactam antibiotics of medicine, have a special role in treating even severe cases of bacterial infections such as sepsis, pneumonia, and complicated urinary tract infections, where other antibiotic treatments either fail completely or the infection is so severe that there is no time to take chances (Caliskan-Aydogan and Alocilja, 2023; Tanveer et al., 2024c). However, the use of these broad-spectrum β-lactam drugs, including imipenem, meropenem, ertapenem, and doripenem, is carefully controlled to slow the emergence and spread of carbapenem-resistant bacteria and to preserve their effectiveness for critical clinical situations (Aurilio et al., 2022; Codjoe and Donkor, 2017; Paudel et al., 2024). Despite these control measures, carbapenem-resistant bacteria have been reported with growing frequency since the early 2000s, initially recognized predominantly in healthcare environments but now detected across a much wider range of non-clinical settings, including livestock and poultry farms, companion animals, wildlife, food and seafood, aquaculture systems, wastewater, surface waters, and other environmental reservoirs (Bonardi and Pitino, 2019; Yigit et al., 2001).

Bacteria can survive carbapenem exposure through resistance mechanisms that reduce or prevent antibiotic activity. One of the most common mechanisms involves the acquisition of carbapenemase-encoding genes such as *bla*OXA, *bla*NDM, *bla*KPC, and *bla*VIM, which produce enzymes capable of hydrolyzing carbapenem antibiotics before they can exert their antibacterial effect (Ghaith et al., 2019; Hamzaoui et al., 2018). Many of these resistance genes are carried on mobile genetic elements, particularly plasmids, which facilitate horizontal dissemination between bacterial populations, while clonal expansion enables their vertical maintenance (Dotto et al., 2014; VinodhKumar et al., 2021). As a consequence, AMR can emerge and spread even in populations with no direct exposure to these antibiotics. Enterobacteriaceae, a large family of gut-associated bacteria, are particularly important in this context because of their widespread presence in humans, animals, and environmental reservoirs such as sewage, wastewater, and contaminated food (Moreira de Gouveia et al., 2024). These bacteria are also highly capable of acquiring and spreading resistance genes. Members of this family are responsible for a wide range of human infections, including urinary tract infections, gastroenteritis, pneumonia, and sepsis (Briongos-Figuero et al., 2012; Díez de Los Ríos et al., 2025; Motarjemi and Adams, 2006). As a result, the emergence of carbapenem-resistant Enterobacteriaceae (CRE) has made the treatment of these infections increasingly difficult and is associated with higher risks of complications and mortality.

Beyond clinical settings, growing evidence suggests that environmental and ecological factors also contribute to the dissemination of CRE and other resistant bacteria (Gross et al., 2022; Tanveer et al., 2024c). In particular, human activities such as urban expansion and land-use change have potentially increased opportunities for contact among wildlife, livestock, and humans, thereby increasing opportunities for resistant bacteria to spread across species (Jori et al., 2021; Tanveer et al., 2025). Wildlife–livestock and wildlife–human interfaces have similarly been recognized as potential routes for exchange of antimicrobial-resistant bacteria and resistance determinants, although the magnitude and direction of transmission remain poorly characterized (Gortázar et al., 2007; Jori et al., 2021). To better understand these patterns across diverse study settings, systematic review and meta-analysis can provide a quantitative synthesis of available evidence across studies while allowing between-study heterogeneity to be assessed (Basile et al., 2025; Haidich, 2010). Previous systematic reviews and meta-analyses have synthesized evidence on the occurrence of CRE across human, livestock, and environmental sources globally (Tuhamize and Bazira, 2024), yet these analyses have concentrated almost exclusively on clinical and agricultural contexts. The occurrence and characteristics of CRE recovered from wildlife have received comparatively little systematic attention. We therefore conducted a systematic review and meta-analysis to provide a comprehensive global synthesis of CRE recovered from wildlife, focusing on the phenotypic occurrence of carbapenem resistance among recovered Enterobacteriaceae isolates, their species distribution, carbapenemase-encoding genes, sequence types, and ecological and geographic patterns within a One Health framework. The primary objective was to characterize the occurrence and molecular features of CRE among Enterobacteriaceae isolates recovered from wildlife-associated samples, rather than to estimate CRE carriage prevalence at the wildlife population level.

## 2 Research methodology

### 2.1 Study design and reporting framework

In this study, a systematic review and meta-analysis were conducted to estimate the pooled CRE-positive proportion among recovered Enterobacteriaceae isolates from wildlife-associated samples. The study design and reporting process followed the guidelines of Preferred Reporting Items for Systematic Reviews and Meta-Analyses (PRISMA; Page et al., 2021; Supplementary Table 1). The research question was developed using the PECO (Population, Exposure, Comparison, and Outcome) framework, in which the population (P) included wildlife species such as mammals, birds, and reptiles sampled either in natural habitats or wildlife-associated facilities, including rehabilitation or rescue centers. The exposure (E) was defined as the wildlife-associated sampling context from which Enterobacteriaceae isolates were recovered. Because the objective of this study was to estimate a pooled CRE-positive proportion rather than to compare exposed and unexposed groups, a comparison group (C) was not considered applicable. The outcome (O) was the proportion of recovered Enterobacteriaceae isolates classified as carbapenem-resistant. Eligible Enterobacteriaceae included *Escherichia coli*, *Klebsiella* spp., *Citrobacter* spp., *Enterobacter* spp., and other relevant genera reported in the included studies.

### 2.2 Literature search strategy

A systematic literature search was performed using three electronic databases (PubMed, Scopus, and Web of Science) to identify relevant studies published between January 1, 2000, and July 25, 2025. No restrictions were applied regarding language or geographic region. Search terms were developed using combinations of keywords related to carbapenem resistance, Enterobacteriaceae, and wildlife, linked with Boolean operators and truncation symbols. The search string included the following terms: (carbapenem* OR imipenem OR meropenem OR ertapenem OR doripenem OR “carbapenem-resistant” OR CRE OR carbapenemase*) AND (Enterobacteriaceae OR Enterobacterales OR Enterobacter* OR “*Escherichia coli*” OR Klebsiella* OR “*Klebsiella pneumoniae*” OR Citrobacter*) AND (wildlife OR “wild animal*” OR “free-living” OR feral OR bird* OR mammal* OR bat* OR rodent* OR raptor* OR rehabilitation OR “rescue center*”). The search strategy was adapted to the syntax and search conventions of each database while retaining the same core concepts and search terms. All references were stored and managed using the reference management software EndNote, and duplicate records were removed after import. To reduce the possibility of missing relevant studies, backward citation tracking was additionally conducted using the reference lists of eligible articles.

### 2.3 Eligibility and exclusion criteria

Studies were included if they met the following criteria: (1) reported carbapenem-resistant Enterobacteriaceae in wildlife; (2) collected samples directly from wildlife species in natural habitats or wildlife-associated facilities; (3) evaluated phenotypic carbapenem resistance using antimicrobial susceptibility testing methods, such as disk diffusion or minimum inhibitory concentration (MIC) testing; and (4) provided sufficient data to calculate the CRE-positive proportion, including the total number of recovered Enterobacteriaceae isolates tested for carbapenem resistance and the number of carbapenem-resistant Enterobacteriaceae isolates. Studies were excluded if wildlife samples were not used, Enterobacteriaceae were not investigated, phenotypic carbapenem resistance was not evaluated, or sufficient information to calculate the CRE-positive proportion was unavailable. Titles and abstracts were independently screened by two reviewers according to the predefined eligibility criteria, followed by full-text evaluation of potentially eligible studies.

### 2.4 Data extraction

To ensure consistent data organization and analysis, data extraction was performed independently by two researchers using a pre-structured Microsoft Excel spreadsheet. Any disagreements during the extraction process were resolved through discussion until consensus was reached. The extracted information included author name, publication year, sampling period, country of study, wildlife species, sample type, sampling environment, antimicrobial susceptibility testing methods, identified Enterobacteriaceae species, total number of wildlife samples, total number of recovered Enterobacteriaceae isolates tested for carbapenem resistance, number of carbapenem-resistant Enterobacteriaceae isolates, tested carbapenem agents, detected carbapenemase-encoding genes, and reported sequence types. Phenotypic carbapenem resistance results were used to define CRE-positive isolates for the meta-analysis, whereas detected carbapenemase-encoding genes and reported sequence types were extracted separately as molecular characteristics of the resistant isolates. In studies containing more than one trial or subgroup (e.g., different wildlife species, carbapenem agent, sample types, or locations), data from each trial were extracted and recorded separately to ensure accuracy. When multiple distinct Enterobacteriaceae isolates were recovered from the same animal, each isolate was retained as an individual observation. In addition, when the same isolates were tested against multiple carbapenem agents, the results for each carbapenem agent were retained as separate isolate–carbapenem observations and were combined for calculation of the overall CRE-positive proportion.

### 2.5 Risk of bias assessment

The risk of bias of the selected studies was assessed independently by three reviewers using the Joanna Briggs Institute (JBI) critical appraisal checklist for prevalence studies. The JBI prevalence checklist was selected because it provides relevant appraisal domains for proportion-based observational studies, including sampling, measurement validity, sample-size adequacy, and statistical analysis (Porritt et al., 2014). The original JBI checklist contains nine assessment items; however, in the present study, eight of the nine risk-of-bias (RoB) criteria were evaluated because the response-rate criterion was not considered applicable to the predominantly non-participant-based wildlife sampling designs included in this review. The evaluated criteria included the study population, sampling methods, clarity of study subject description, accuracy and reliability of diagnostic methods, data collection approach, adequacy of sample size, and suitability of statistical methods. The studies were evaluated for potential sources of bias and categorized as having low, high, or unclear risk of bias. Disagreements between reviewers were resolved through discussion and consensus.

### 2.6 Data synthesis and statistical analysis

All statistical analyses were performed using R software (version 4.4.3; R Studio version 2024) (Balduzzi et al., 2019). Meta-analyses, meta-regression, sensitivity analyses, and graphical visualizations were conducted using the “meta,” “metafor,” and “ggplot2” packages (Alboukadel, 2018; Viechtbauer, 2010). The CRE-positive proportion was calculated as the number of carbapenem-resistant Enterobacteriaceae isolates divided by the total number of recovered Enterobacteriaceae isolates tested for carbapenem resistance in each study. Therefore, the pooled estimate represents an isolate-level CRE-positive proportion and should not be interpreted as the proportion of wild animals or wildlife samples carrying CRE.


CRE-positive⁢proportion=

$$\frac{\mathrm{Number}\,\mathrm{of}\,\mathrm{carbapenem}\,\mathrm{resistant}\,\mathrm{isolates}}{\mathrm{Total}\,\mathrm{number}\,\mathrm{of}\,\mathrm{recovered}\,\mathrm{Enterobacteriaceae}\,\mathrm{isolates}}$$

Because substantial variability among studies was anticipated due to differences in CRE species, carbapenem agent tested, wildlife taxa, geographical location, sample type, sampling environment, susceptibility testing, sample size, and publication year, pooled CRE-positive proportions were calculated using a random-effects model. This model accounts for both within-study and between-study variability and is appropriate for synthesizing proportions from heterogeneous studies. The Freeman–Tukey double-arcsine transformation was used because the dataset included sparse denominators and extreme proportions, including 100% estimates, and this transformation allows inclusion of extreme proportions while stabilizing variances (Abdulmajeed et al., 2025), and was expressed as:


$$P\,F\,T=\frac{1}{2}\,\left[a\,r\,c\,s\,i\,n\,\left(\sqrt{\frac{x}{n+1}}\right)+a\,r\,c\,s\,i\,n\,\left(\sqrt{\frac{x+1}{n+1}}\right)\right]$$

where *x* is the number of carbapenem-resistant Enterobacteriaceae isolates, and *n* is the total number of recovered Enterobacteriaceae isolates tested for carbapenem resistance. This transformation allowed inclusion of studies with extreme proportions without excluding them or depending strongly on continuity corrections.

Pooled estimates and corresponding 95% confidence intervals (CIs) were subsequently back-transformed and presented as percentages for ease of interpretation (Barendregt et al., 2013; Tanveer et al., 2024a). Between-study variance (τ^2^) was estimated using restricted maximum-likelihood (REML) estimation, and the Knapp–Hartung adjustment was applied to obtain more conservative confidence intervals (Tanveer et al., 2024b; van Aert and Jackson, 2019). Statistical heterogeneity among studies was assessed using Cochran’s Q test, τ^2^, and the *I*^2^ statistic. Heterogeneity was considered substantial when the *Q*-test yielded a *p*-value < 0.05, and *I*^2^ exceeded 50%. Forest plots were generated to visually summarize pooled CRE-positive proportions across studies. To explore potential sources of heterogeneity, subgroup analyses and meta-regression analyses were performed using *a priori*-selected moderators, including CRE species group (*Escherichia coli*, *Klebsiella* spp., and other Enterobacteriaceae genera), carbapenem agent, wildlife taxonomic order, study location by continent, sample type, susceptibility testing, publication year, sampling environment, and sample size. Potential publication bias was assessed visually using funnel plots and statistically using Egger’s regression test when sufficient studies were available. Where asymmetry was suspected, the Duval and Tweedie trim-and-fill method was applied as a sensitivity analysis to evaluate the potential influence of missing studies on the pooled CRE-positive proportion (Duval and Tweedie, 2000). Leave-one-out sensitivity analysis and influence diagnostics were also performed to assess whether any individual study exerted a disproportionate influence on the pooled estimate.

## 3 Results

### 3.1 Search results

A total of 6,988 records were identified through database searches and other sources. After removal of 721 duplicates, 6,267 records remained for title and abstract screening. Of these, 5,526 were excluded after initial screening, leaving 741 articles for full-text assessment. Following full-text review, 722 articles were further excluded for the following reasons: carbapenem resistance testing was not performed (*n* = 701), review articles (*n* = 7), samples not collected from wild animals (*n* = 6), non-prevalence studies (*n* = 7), and letters to the editor (*n* = 1). Finally, 19 studies met the eligibility criteria and were included in the systematic review and meta-analysis. The study selection process is presented in Figure 1A.

**FIGURE 1:**
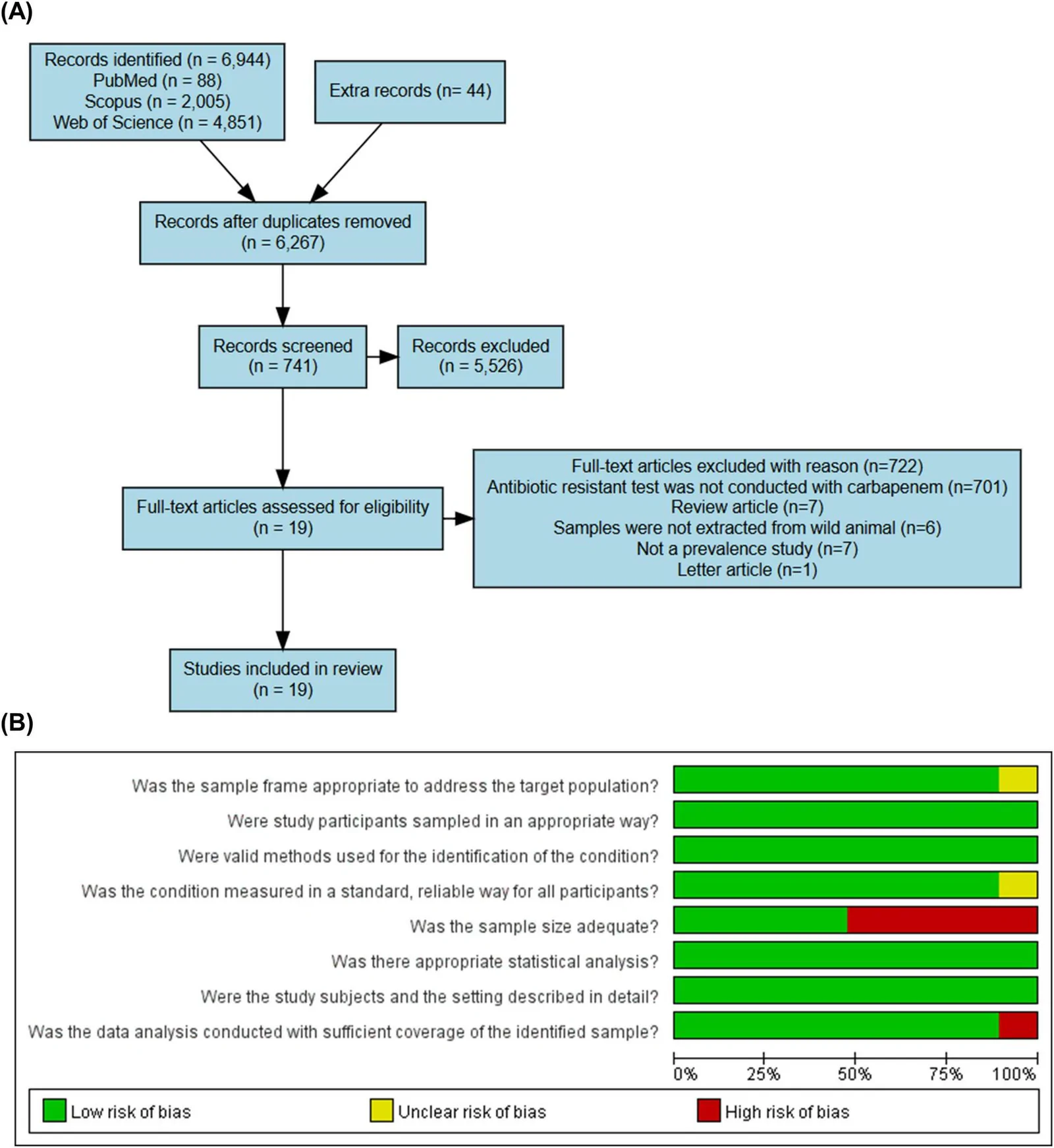
**(A)** PRISMA flow diagram showing the study selection process for the systematic review and meta-analysis of carbapenem-resistant Enterobacteriaceae in wild animals. **(B)** Risk-of-bias assessment of eligible studies included in the systematic review and meta-analysis, evaluated using the Joanna Briggs Institute (JBI) critical appraisal checklist for prevalence studies.

### 3.2 Risk of bias assessment

The methodological quality of the included studies was assessed using the JBI critical appraisal checklist for prevalence studies, and the overall results are summarized in Figure 1B. Among the 19 included studies, three were classified as having a low risk of bias across all evaluated domains. Most studies demonstrated adequate methodological quality regarding sample representativeness and sampling procedures. Seventeen studies identified wildlife samples at the species level, whereas two studies reported only genus-level identification, resulting in lower specificity of the target population description. In addition, the majority of studies collected fecal, rectal swab, or cloacal swab samples directly from wildlife using appropriate sampling approaches. Based on the JBI sample size criterion (95% confidence interval, expected prevalence = 0.1, margin of error = 0.05), the minimum recommended sample size was estimated to be 140. Nine studies met or exceeded this threshold and were therefore considered to have a lower risk of bias related to sample size adequacy, whereas ten studies included fewer than 140 samples. All studies provided sufficient information regarding laboratory procedures or study settings. Sixteen studies adequately characterized the isolated Enterobacteriaceae species, while three studies lacked detailed bacterial characterization. Antimicrobial susceptibility testing was performed using standardized methods, including minimum inhibitory concentration (MIC) testing or disk diffusion assays, across all studies. Although most studies clearly described bacterial isolation and culture procedures, methodological details were incomplete in three studies. Carbapenem resistance and bacterial characterization were assessed using established microbiological and molecular methods across studies. However, statistical analysis methods were adequately described in only seven studies, whereas the remaining studies provided limited information regarding analytical procedures, resulting in a higher or unclear risk of bias in this domain.

### 3.3 Study characteristics

The 19 studies included in the meta-analysis investigated CRE isolated from diverse wildlife species across multiple geographic regions (Ahlstrom et al., 2019, 2022; Athanasakopoulou et al., 2022; Bachiri et al., 2018; Ballash et al., 2022; Bouaziz et al., 2018; de Sousa et al., 2022; Gharout-Sait et al., 2019; Ju et al., 2025; Kabali et al., 2021; Kumar et al., 2021; Liao et al., 2019; Loucif et al., 2022; Selmi et al., 2022; Tarabai et al., 2021; Vergara et al., 2017; VinodhKumar et al., 2021; Vittecoq et al., 2017; Yan et al., 2024). The detailed characteristics of the included studies are summarized in Figure 2 and Supplementary Table 2. Reported CRE species included *Escherichia coli*, *Klebsiella pneumoniae*, *Klebsiella quasipneumoniae*, *Enterobacter cloacae*, and *Citrobacter freundii*. Due to the limited number of reports involving Enterobacteriaceae species other than *E. coli*, isolates were categorized into three groups for analysis: *E. coli*; *Klebsiella* spp. (including *K. pneumoniae* and *K. quasipneumoniae*) and other Enterobacteriaceae genera (including *Citrobacter* and *Enterobacter* spp.). Among the included studies, *E. coli* was the most frequently reported species group (12 studies), followed by *Klebsiella* spp. (9 studies) and other Enterobacteriaceae (3 studies). Three studies reported two Enterobacteriaceae species from the same samples, whereas one study simultaneously identified three species. The studies included a wide range of wildlife hosts, particularly birds and mammals, with samples collected from feces, rectal swabs, cloacal swabs, and tissue-associated specimens from wild, captured, captive, or park-associated animals. Most studies relied on fecal samples for bacterial isolation (14 studies). Based on the reported sampling conditions, study environments were categorized as natural, water-associated, human-associated, captive/rehabilitation-related, and not-reported environments. Water-associated and natural environments were each represented in six studies, whereas four studies were conducted in human-associated environments and three in captive settings. Four studies did not provide detailed environmental information.

**FIGURE 2:**
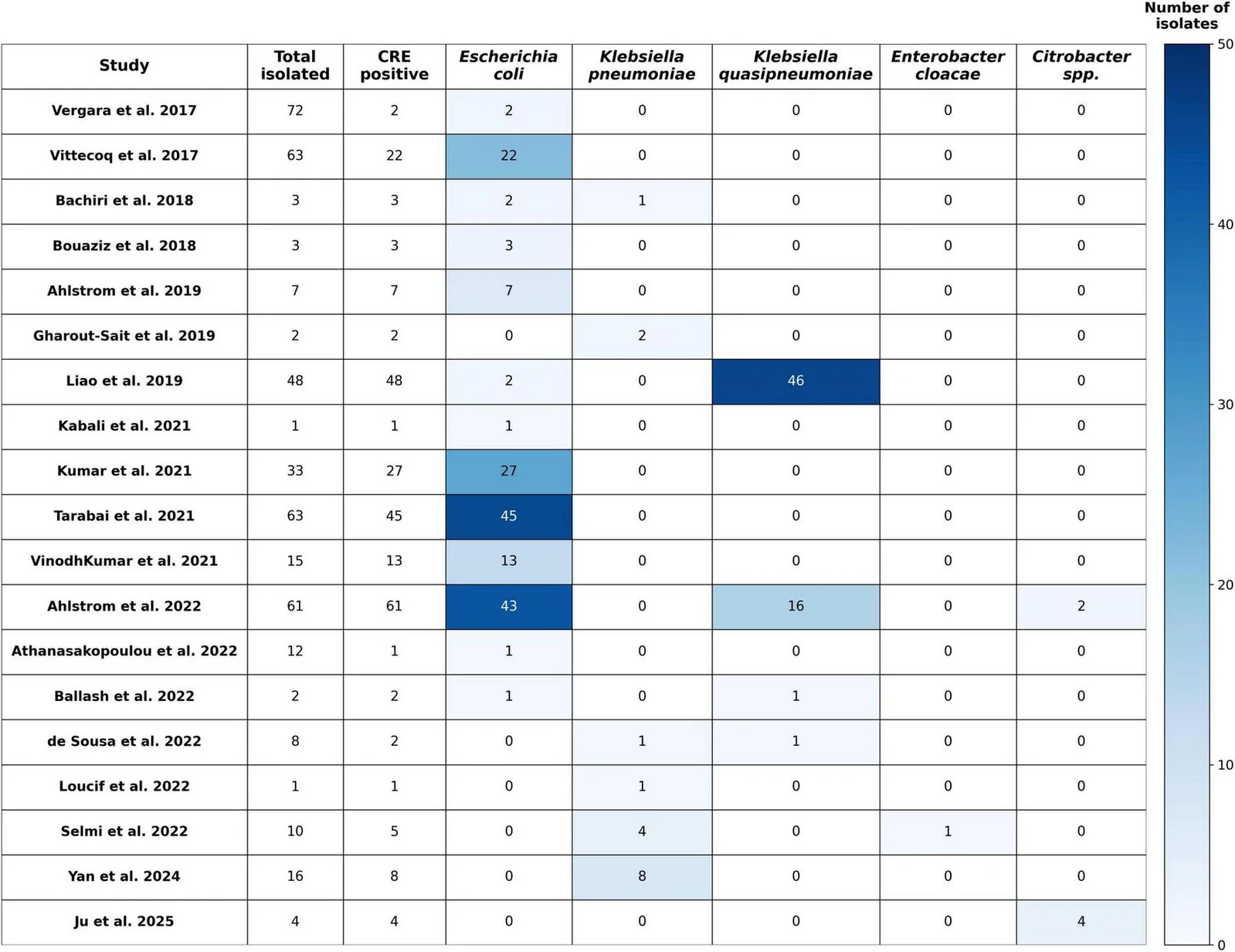
Distribution of carbapenem-resistant Enterobacteriaceae isolated from wildlife across the included studies.

Antimicrobial susceptibility testing was performed using carbapenem agents, including imipenem, ertapenem, and meropenem. Meropenem and imipenem were the most commonly used antibiotics, although several studies evaluated multiple carbapenem agents simultaneously. Laboratory methods varied across studies. Carbapenem resistance was assessed using phenotypic susceptibility testing, including MIC assays and disk diffusion, while culture-based methods and PCR were used for bacterial isolation, identification, and carbapenemase gene detection. The included studies were distributed across multiple continents, with the largest contributions from Asia (China, India, Turkey, Pakistan) and Africa (Algeria, Zambia, Tunisia), each accounting for six studies. Four studies were conducted in Europe (Spain, France, Greece, Ukraine), two in South America (Chile, Brazil), and two in North America (USA), while one study originated from Oceania (Australia). One multicountry study included wildlife samples collected from Turkey, Chile, Spain, Ukraine, and Pakistan. Overall, 12 studies were published after 2020, reflecting the increasing recent attention toward CRE dissemination in wildlife. Several clinically important carbapenemase-encoding genes were identified among the isolates. OXA-family genes and NDM variants were the most frequently reported resistance determinants, followed by KPC, VIM, and IMP genes. Sequence type information was unavailable in eight studies. Among the reported sequence types, ST38 was the most frequently detected lineage, followed by ST410 and ST297.

### 3.4 Meta-analysis and moderator analysis

The pooled CRE-positive proportion among recovered Enterobacteriaceae isolates was estimated at 74.49% (95% CI: 49.75–94.04), with substantial heterogeneity among studies (*I*^2^ = 96%) (Figure 3). Owing to this high between-study variability, moderator analyses were performed using subgroup analysis across nine *a priori*-selected variables: CRE species, wildlife taxonomic order, carbapenem agent, susceptibility testing method, sample type, sample size, sampling environment, continent, and publication year. Detailed results of subgroup analyses are presented in Supplementary Figures 2–10 and Supplementary Table 3. According to CRE species, the highest pooled CRE-positive proportion was observed among other Enterobacteriaceae genera, including *Citrobacter* and *Enterobacter* spp. (86.46%, 95% CI: 0–100), followed by *Escherichia coli* (76.51%, 95% CI: 45.22–98.45) and *Klebsiella* spp. (72.41%, 95% CI: 28.29–100). No statistically significant subgroup difference was observed across CRE species categories (*p* = 0.89), although heterogeneity remained high in the *E. coli* and *Klebsiella* spp. subgroups (*I*^2^ = 95% for both). Subgroup analysis by wildlife taxonomic order showed that an integrated subgroup of wildlife orders including Ciconiiformes, Chiroptera, Anseriformes, Accipitriformes, Psittaciformes, and Columbiformes, had the highest pooled CRE-positive proportion (92.20%, 95% CI: 36.07–100), followed by Artiodactyla (77.81%, 95% CI: 39.16–100), Charadriiformes (62.35%, 95% CI: 13.30–99.44), and Carnivora (53.81%, 95% CI: 0–100). Differences among wildlife taxonomic orders were not statistically significant (*p* = 0.68). Heterogeneity varied across orders, ranging from low in Artiodactyla (*I*^2^ = 5%) to very high in Charadriiformes and Carnivora (>95%).

**FIGURE 3:**
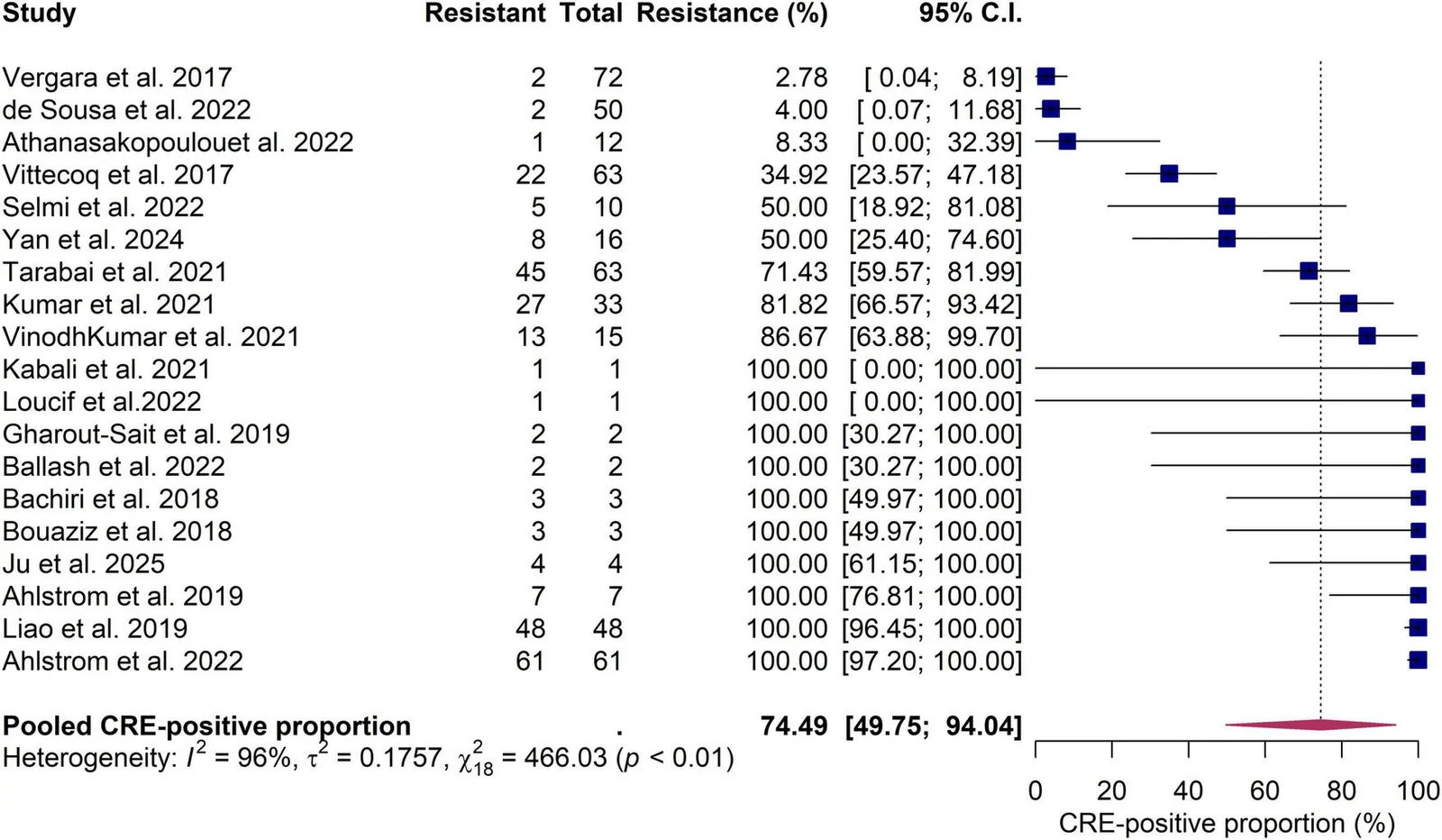
Forest plot showing the pooled CRE-positive proportions among recovered Enterobacteriaceae isolates from wild animals.

Across carbapenem agents, ertapenem showed the highest pooled isolate-level CRE-positive proportion (90.81%, 95% CI: 72.5–100), followed by meropenem (80.69%, 95% CI: 46.26–100) and imipenem (67.73%, 95% CI: 33.55–94.98). Subgroup differences according to carbapenem agent were not statistically significant (*p* = 0.39), while substantial heterogeneity remained within the carbapenem-agent subgroups. Subgroup analysis according to susceptibility testing method showed a pooled CRE-positive proportion of 85.75% (95% CI: 11.33–100) for MIC testing and 69.62% (95% CI: 41.94–92.42) for disk diffusion testing. The subgroup difference was not statistically significant (*p* = 0.54), and substantial heterogeneity remained within both subgroups (*I*^2^ = 99% and 95%, respectively). Sample type-based analysis showed the highest pooled CRE-positive proportion in fecal samples (89.29%, 95% CI: 66.91–100), followed by rectal/cloacal/wound swabs (23.04%, 95% CI: 0–63.97). Overall, subgroup differences by sample type were statistically significant (*p* < 0.01). Sampling environment-based analysis showed that isolate-level CRE-positive proportion varied across different sampling contexts. The highest pooled CRE-positive proportion was observed in water-associated sites (84.49%), followed by captive (79.65%), natural (77.06%), human-associated (72.36%), and not-reported site categories (51.61%). However, differences among sampling environments were not statistically significant (*p* = 0.94), and substantial heterogeneity persisted across most subgroups.

When stratified by sample size, studies with fewer than 30 samples showed a pooled CRE-positive proportion of 76.22% (95% CI: 47.83–97.14), whereas studies with more than 30 samples showed a pooled CRE-positive proportion of 67.88% (95% CI: 5.8–100). The subgroup difference by sample size was not statistically significant (*p* = 0.90), and substantial heterogeneity persisted in both categories. Continent-based analysis showed the highest pooled CRE-positive proportion in Africa (91.53%, 95% CI: 58.25–100), followed by Asia–Pacific (88.33%, 95% CI: 67.15–99.9), the Americas (North and South) (80.45%, 95% CI: 0–100), and Europe (37.35%, 95% CI: 0–100). Subgroup differences by continent were not statistically significant (*p* = 0.39). Finally, subgroup analysis by publication year showed a pooled CRE-positive proportion of 80.11% (95% CI: 27.18–100) in studies published before 2020 and 71.16% (95% CI: 39.74–95.63) in studies published after 2020. The subgroup difference by publication year was not statistically significant (*p* = 0.68), and substantial heterogeneity remained in both periods.

Because substantial heterogeneity persisted across subgroup analyses, univariate meta-regression was further performed to quantify between-study heterogeneity explained by the selected moderators. Detailed results of meta-regression analyses are presented in Supplementary Figures 11–19 and Supplementary Table 3. Sample type was the only moderator showing a statistically significant association in univariate meta-regression (R^2^ = 33.1%, *p* = 0.0029). Continent accounted for 8.31% of the estimated between-study heterogeneity, but its association was not statistically significant (*p* = 0.2013), while the remaining moderators explained little or no heterogeneity. However, substantial residual heterogeneity remained, indicating that the observed variability was not fully explained by any single measured moderator. Therefore, multivariable meta-regression was not performed, as most available moderator variables did not demonstrate meaningful explanatory value in univariate models and inclusion of multiple variables could increase the risk of overfitting. Sensitivity analysis was also performed using leave-one-out analysis and studentized residuals to assess the influence of individual studies on the pooled estimate (Supplementary Figure 1). Vergara et al. (2017) and de Sousa et al. (2022), which reported low CRE-positive proportions of 2/72 and 2/50, respectively, showed relatively stronger influence in the diagnostic analysis. However, these studies were retained because they represented valid data and reflected the variability in CRE occurrence across wildlife-associated studies. Leave-one-out analysis further indicated that the pooled estimate was not driven by any single study.

### 3.5 Geographic distribution of carbapenemase-encoding genes identified in CRE

Wildlife-associated CRE carrying clinically important carbapenemase-encoding genes have been reported across multiple continents, indicating their global distribution (Figure 4). Several major carbapenemase families were identified, including NDM, KPC, OXA-48-like, VIM, and IMP, indicating substantial genetic diversity among wildlife-associated CRE isolates. Among these, NDM-family carbapenemases were frequently reported and were particularly common in Asia, where NDM genes were detected in studies from Pakistan, India, and China. NDM-5 was the most frequently reported subtype in this region, followed by other variants such as NDM-1 and NDM-7. These genes were detected primarily in *Escherichia coli*, *Klebsiella quasipneumoniae*, and *Citrobacter freundii*, indicating the occurrence of NDM-producing Enterobacteriaceae among wildlife-associated isolates in Asia. In Turkey, OXA-48 and KPC-2 were reported in *E. coli* and *Citrobacter* spp., suggesting additional diversity in carbapenemase-mediated resistance within the region.

**FIGURE 4:**
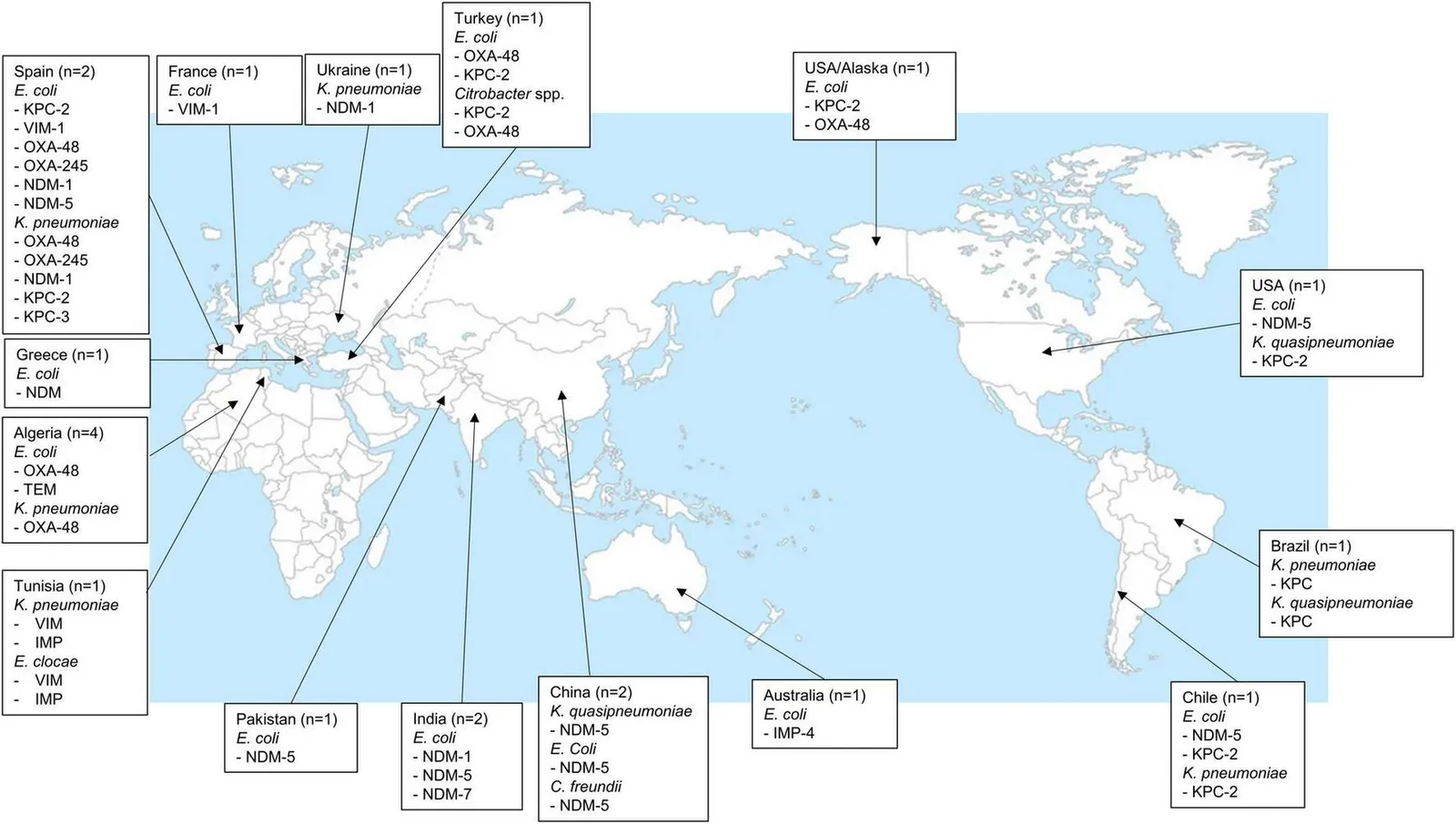
Global distribution of reported carbapenemase-encoding genes identified in carbapenem-resistant Enterobacteriaceae isolated from wild animals.

In Europe, carbapenemase-encoding gene distribution was diverse, with NDM, KPC, OXA-48-like, and VIM-family genes reported across different countries. Spain showed the broadest diversity, with detection of KPC-2, KPC-3, VIM-1, OXA-48, OXA-245, NDM-1, and NDM-5 in *E. coli* and *Klebsiella pneumoniae*. VIM-1 was also reported in France, while NDM genes were identified in Ukraine and Greece. These findings suggest that wildlife-associated CRE in Europe harbor multiple carbapenemase families rather than a single predominant carbapenemase group. Studies from North and South America mainly reported KPC-lineage carbapenemases, particularly KPC-2, which was detected in isolates from the USA, Brazil, Chile, and USA/Alaska. However, other carbapenemase-encoding genes were also reported, including NDM-5 in the USA and Chile and OXA-48 in USA/Alaska. This indicates that although KPC enzymes were common in the Americas, additional carbapenemase families are also present among wildlife-associated Enterobacteriaceae in this region.

In Africa, OXA-48 was reported in studies from Algeria, where it was detected in *E. coli* and *K. pneumoniae*. However, the Figure 4 also shows VIM and IMP genes in Tunisia, detected in *K. pneumoniae* and *Enterobacter cloacae*. Therefore, the African data indicate the presence of multiple carbapenemase families, with OXA-48 being prominent in Algeria but not exclusive across the continent. In Oceania, the only included study identified IMP-4-producing *E. coli* in Australia, demonstrating the presence of carbapenemase-producing wildlife-associated isolates in the region despite the limited number of available reports. Overall, the geographic variation observed in carbapenemase-encoding gene distribution suggests that the epidemiology of carbapenem-resistant Enterobacteriaceae in wildlife may reflect regional antimicrobial resistance patterns documented in human, animal, and environmental settings. The observed distribution highlights the global occurrence of clinically important carbapenemase-encoding genes in wildlife-associated CRE, including NDM-dominant patterns in parts of Asia, diverse carbapenemase profiles in Europe, frequent KPC detection in the Americas, OXA-48 occurrence in North Africa, and IMP detection in Tunisia and Australia.

### 3.6 Geographic distribution of sequence types identified in CRE

A diverse range of multilocus sequence typing (MLST) sequence types was identified among carbapenem-resistant Enterobacteriaceae isolated from wild animals across multiple geographic regions (Figure 5). Most reported sequence types were associated with *Escherichia coli*, although several *Klebsiella pneumoniae*, *Klebsiella quasipneumoniae*, and *Citrobacter freundii* lineages were also identified. Among the reported sequence types, *E. coli* ST38 was one of the most recurrent lineages and was identified in Spain, Algeria, Turkey, and the USA/Alaska. ST410 was detected in Spain and USA/Alaska, whereas ST744 was identified in Spain and Chile. These findings indicate that a limited number of *E. coli* lineages were shared across geographically distant regions, although most sequence types were reported in single countries. In Asia, sequence type diversity was particularly evident in China, where multiple *K. pneumoniae* lineages were reported, including ST1558, ST4007, ST1319, ST2541, ST2729, ST894, ST309, and ST2671. Several *K. quasipneumoniae* sequence types were also identified in China, including ST35, ST1697, ST2582, ST2583, and ST2584, along with *C. freundii* ST1124. Additional Asian sequence types included *E. coli* ST167 in Pakistan, *E. coli* ST297 in India, and *E. coli* ST38 in Turkey.

**FIGURE 5:**
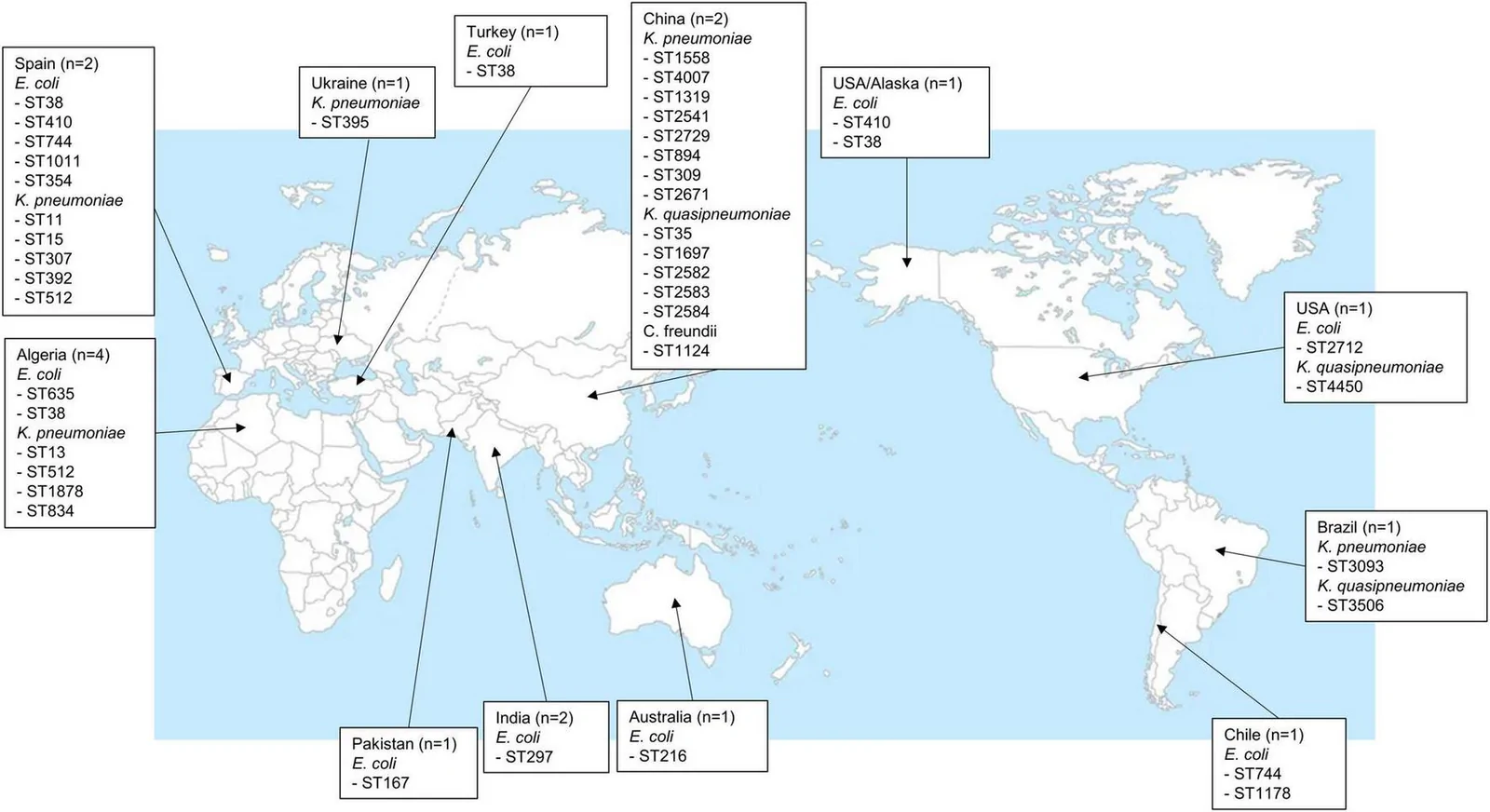
Global distribution of reported sequence types (STs) identified among carbapenem-resistant Enterobacteriaceae isolated from wild animals.

In Europe, Spain showed the greatest MLST diversity, with *E. coli* ST38, ST410, ST744, ST1011, and ST354, as well as *K. pneumoniae* ST11, ST15, ST307, ST392, and ST512. Ukraine reported *K. pneumoniae* ST395. These findings suggest that wildlife-associated carbapenem-resistant Enterobacteriaceae in Europe include both globally recognized *E. coli* lineages and diverse *K. pneumoniae* sequence types. In Africa, Algeria reported multiple sequence types, including *E. coli* ST635 and ST38, as well as *K. pneumoniae* ST13, ST512, ST1878, and ST834. In North America, USA/Alaska reported *E. coli* ST410 and ST38, while the USA reported *E. coli* ST2712 and *K. quasipneumoniae* ST4450. In South America, Brazil reported *K. pneumoniae* ST3093 and *K. quasipneumoniae* ST3506, whereas Chile reported *E. coli* ST744 and ST1178. In Oceania, the only included study from Australia reported *E. coli* ST216. Overall, the observed MLST distribution demonstrates substantial genetic heterogeneity among wildlife-associated carbapenem-resistant Enterobacteriaceae. Although recurrent lineages such as *E. coli* ST38, ST410, and ST744 were detected in more than one geographic region, most sequence types appeared to be country- or study-specific. The detection of diverse *E. coli*, *K. pneumoniae*, *K. quasipneumoniae*, and *C. freundii* lineages across continents highlights that wildlife-associated samples can contain genetically diverse carbapenem-resistant Enterobacteriaceae.

### 3.7 Publication bias

Publication bias was assessed to determine whether the overall pooled CRE-positive proportion may have been influenced by selective publication, missing studies, or small-study effects (Figure 6). Visual inspection of the funnel plot showed asymmetry, suggesting the possible presence of publication bias or small-study effects. However, Egger’s regression test did not provide statistically significant evidence of funnel plot asymmetry (*t* = 1.6924, *p* = 0.1088). To further evaluate the potential impact of asymmetry, the trim-and-fill method was applied, which estimated four potentially missing studies on the left side of the funnel plot. After adjustment, the pooled CRE-positive proportion decreased from 74.49% (95% CI: 49.75–94.04) to 59.64% (95% CI: 35.1–82.38), indicating that the original estimate may have been influenced by funnel plot asymmetry. Nevertheless, substantial heterogeneity persisted after adjustment (*I*^2^ = 95.52%), suggesting that publication bias or small-study effects did not fully explain the between-study variability. Overall, the funnel plot asymmetry and trim-and-fill analysis suggest possible small-study effects; however, these findings should be interpreted cautiously because the number of included studies was limited, Egger’s test was non-significant, and substantial heterogeneity persisted after adjustment. Therefore, the observed asymmetry may reflect methodological and ecological heterogeneity in addition to, or instead of, publication bias.

**FIGURE 6:**
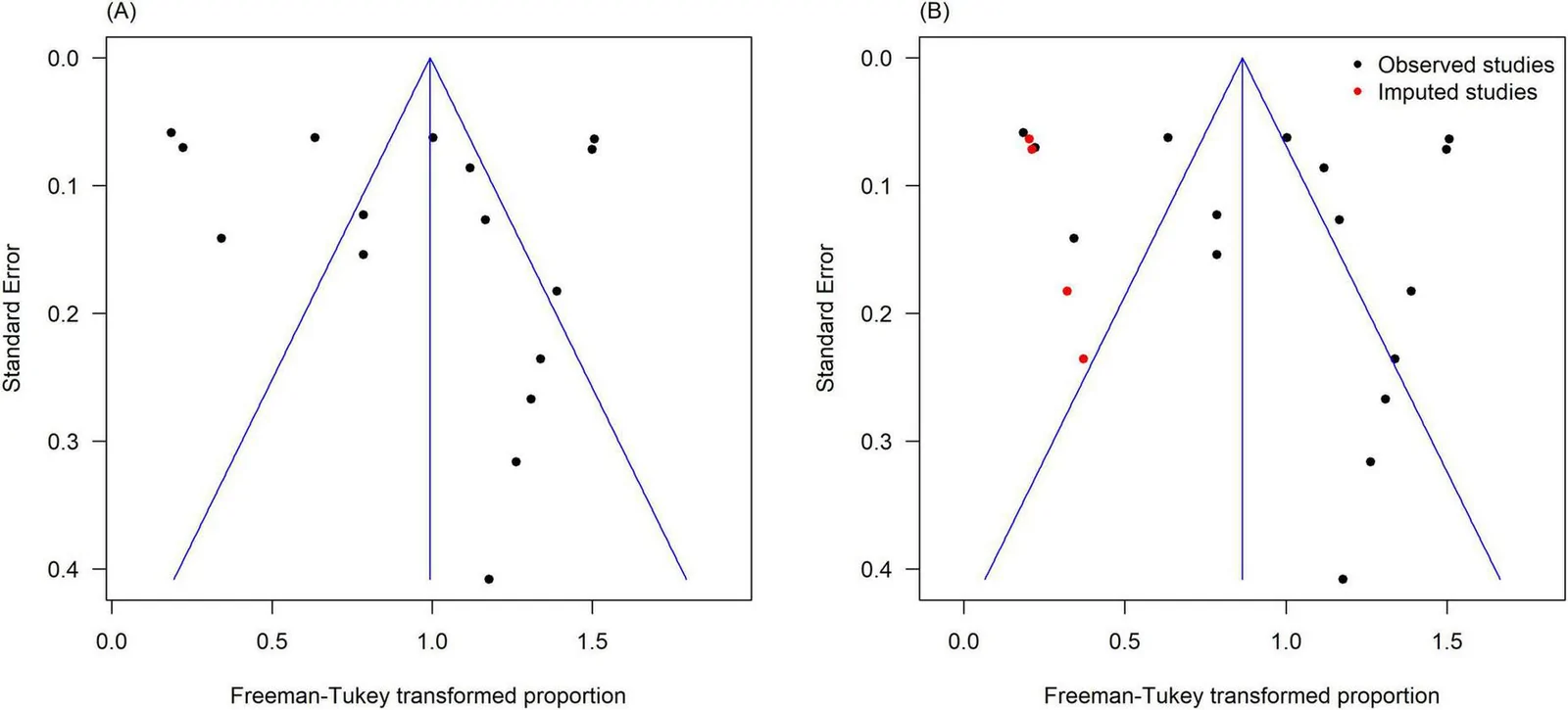
Funnel plots assessing publication bias in studies reporting CRE-positive proportions among recovered Enterobacteriaceae isolates from wild animals. **(A)** Funnel plot of the observed studies. **(B)** Funnel plot after application of the trim-and-fill method, showing observed and imputed studies.

## 4 Discussion

Carbapenem-resistant Enterobacteriaceae (CRE) are increasingly recognized as a major global public health concern because carbapenems are considered last-resort antimicrobials for the treatment of severe multidrug-resistant Gram-negative bacterial infections (Ma et al., 2023). The detection of CRE in wildlife-associated samples, therefore, raises important concerns regarding environmental contamination, interspecies transmission, and the broader ecological spread of antimicrobial resistance (Ramírez-Castillo et al., 2023). This study synthesized available evidence regarding the isolate-level CRE-positive proportion, molecular characteristics, and geographic distribution of CRE recovered from wildlife-associated samples and demonstrated that clinically important carbapenem-resistant bacteria and carbapenemase-encoding genes are widely distributed across multiple continents. Because the analysis was based on recovered Enterobacteriaceae isolates, these estimates should not be interpreted as wildlife-level CRE carriage prevalence. Among the bacterial groups included in this meta-analysis, CRE-positive proportions were descriptively high across *Escherichia coli*, *Klebsiella* spp., and other Enterobacteriaceae genera, including *Citrobacter* and *Enterobacter* spp. Although other Enterobacteriaceae showed the highest pooled estimate, differences among species groups were not statistically significant (*p* = 0.89), and this estimate should be interpreted cautiously because it was based on fewer studies and had wide uncertainty (Supplementary Table 3). The frequent detection of carbapenem-resistant *E. coli* remains epidemiologically important because *E. coli* commonly serves as both an intestinal commensal organism and an important reservoir of antimicrobial resistance genes in humans, animals, and environmental systems. Its recovery from wildlife may reflect ecological adaptability and the ability to acquire resistance determinants through horizontal gene transfer (Aljohni et al., 2025; Foster-Nyarko and Pallen, 2022). Similar patterns have been reported in studies investigating antimicrobial-resistant Enterobacteriaceae in migratory birds, aquatic environments, and urban wildlife, where *E. coli* frequently predominated among resistant isolates (Ramírez-Castillo et al., 2023; Sabença et al., 2024). Overall, these findings indicate that CRE are not restricted to clinical or hospital environments alone, but are also present in environmental and wildlife-associated ecosystems. The increasing number of studies published after 2020 further suggests growing scientific concern regarding the role of wildlife in antimicrobial resistance dissemination. However, the subgroup analysis did not show a statistically significant difference in CRE-positive proportions between studies published before and after 2020 (*p* = 0.68) (Athanasakopoulou et al., 2022; Di Francesco et al., 2026).

Descriptive variation in CRE-positive proportions was observed across continents, with the highest pooled estimate in Africa, followed by Asia–Pacific, the Americas, and Europe (Supplementary Table 3). However, the subgroup difference by continent was not statistically significant (*p* = 0.39). The observed descriptive variation may reflect differences in antimicrobial usage patterns, healthcare-associated dissemination, wastewater management, environmental contamination, wildlife ecology, and surveillance intensity. Previous studies conducted in the United States, Australia, and Latin America have similarly documented increasing dissemination of carbapenemase-producing Gram-negative bacteria in human and environmental settings (Australian Government Department of Health, 2023; Pillonetto et al., 2025; Rhodes et al., 2019). Since carbapenems are primarily reserved for severe clinical infections, increased carbapenem consumption may contribute to selective pressure favoring resistant strains (Tamma et al., 2022). Nevertheless, antibiotic usage alone is unlikely to fully explain the observed geographic patterns. Environmental contamination pathways, wildlife migration, anthropogenic ecosystem disturbance, and ecological persistence of resistant bacteria probably also contribute substantially to CRE dissemination (Laborda et al., 2022; Lee et al., 2022). The high heterogeneity observed across subgroup analyses likely reflects the considerable diversity among included studies regarding wildlife taxa, geographic location, sample type, diagnostic method, carbapenem agent, sample size, and publication year. Differences in antimicrobial susceptibility testing protocols, bacterial isolation methods, and molecular detection techniques may also have contributed to variability in CRE-positive proportion estimates (Fluit et al., 2001; Verma and Balekar, 2023). In addition, several subgroup estimates were based on a limited number of study trials, resulting in wide confidence intervals and reduced precision. No statistically significant subgroup differences were observed for Enterobacteriaceae species, wildlife taxonomic order, carbapenem agent, susceptibility testing method, sample size, sampling environment, continent, or publication year. In contrast, sample type was the only variable showing a statistically significant subgroup difference and was also the only statistically significant moderator in univariate meta-regression (R^2^ = 33.1%, *p* = 0.0029). Continent accounted for 8.31% of the estimated between-study heterogeneity, but this association was not statistically significant (*p* = 0.2013). However, substantial residual heterogeneity remained, suggesting that the observed variability was multifactorial and not fully explained by any single measured moderator. Overall, these findings highlight the need for more standardized surveillance methodologies and more detailed reporting in future wildlife antimicrobial resistance studies.

An important methodological consideration is that the CRE-positive proportion among recovered Enterobacteriaceae isolates is inherently influenced by the bacterial isolation procedures used in the original studies. In studies employing selective or enrichment culture methods designed to facilitate recovery of antimicrobial-resistant bacteria, susceptible organisms may be suppressed while resistant organisms are preferentially recovered. Consequently, a high CRE-positive proportion among recovered isolates may partly reflect the laboratory selection strategy rather than the underlying frequency of CRE in the wildlife population. Differences in culture and enrichment procedures across studies may also have contributed to the substantial between-study heterogeneity observed in this meta-analysis. Therefore, the pooled estimate should be interpreted specifically as the proportion of recovered Enterobacteriaceae isolates classified as carbapenem-resistant under the laboratory procedures of the included studies, rather than as an estimate of CRE carriage prevalence in wildlife populations.

A descriptively high CRE-positive proportion was observed in water-associated environments; however, differences among sampling environments were not statistically significant (*p* = 0.94) (Supplementary Table 3). Similar observations have been reported in studies investigating wastewater systems, rivers, aquatic ecosystems, and sewage-associated environments (Galler et al., 2014; Guo et al., 2023; Teng et al., 2021; Zurfluh et al., 2017). Wastewater treatment plants may not completely eliminate resistant bacteria or resistance genes, thereby facilitating their persistence and dissemination into surrounding ecosystems (Zhu et al., 2025). Wildlife species inhabiting or frequently visiting aquatic environments may therefore experience repeated exposure to antimicrobial-resistant bacteria originating from anthropogenic activities (Thibodeau et al., 2024). This interpretation is also consistent with the descriptively elevated CRE-positive proportion observed among Charadriiformes, a taxonomic order that includes many water-associated and migratory bird species, although differences among wildlife taxonomic orders were not statistically significant (Supplementary Table 3). Many migratory bird species inhabit coastal environments, wetlands, or wastewater-associated habitats and travel long distances across continents, potentially facilitating exposure to, carriage of, or movement of resistant bacteria and resistance genes across environments. Previous studies have similarly identified multidrug-resistant and carbapenemase-producing *E. coli* in gulls and other migratory birds inhabiting human-impacted aquatic environments (Ahlstrom et al., 2019; Masarikova et al., 2024; Zeballos-Gross et al., 2021). Although direct transmission pathways remain difficult to establish, the repeated detection of similar resistant bacteria in humans, wildlife, and environmental samples supports the interconnected nature of these ecosystems within a One Health framework (Pandey et al., 2024). The comparatively limited number of studies investigating *Citrobacter* spp. and *Enterobacter* spp. demonstrates an important knowledge gap regarding non-*E. coli* Enterobacteriaceae in wildlife (Supplementary Table 3). Despite the limited number of available studies, the detection of carbapenem-resistant *Citrobacter* and *Enterobacter* isolates remains epidemiologically important because these genera are increasingly recognized as opportunistic pathogens capable of acquiring clinically relevant carbapenemase-encoding genes (Babiker et al., 2020). The wide confidence intervals and high heterogeneity observed in this subgroup further emphasize the need for additional surveillance studies focusing on less frequently investigated Enterobacteriaceae species (Supplementary Table 3).

The geographic distribution of carbapenemase-encoding genes identified in this study is further consistent with possible environmental dissemination and broad circulation of clinically important resistance determinants (Figure 4). Several major carbapenemase-encoding gene families were identified, including *bla*_*NDM*_, *bla*_*KPC*_, *bla*_*OXA–48*_, *bla*_*VIM*_, and *bla*_*IMP*_. Among these, *bla*_*NDM*_ genes were frequently reported and were particularly common in Asia, whereas *bla*_*KPC*_ genes were commonly detected in North and South America. In Africa, *bla*_*OXA–48*_ was detected in Algeria, while *bla*_*VIM*_ and *bla*_*IMP*_ were reported in Tunisia, indicating that multiple carbapenemase-encoding gene families are present among wildlife-associated Enterobacteriaceae on the continent. Similarly, Europe showed a diverse carbapenemase profile, with detection of *bla*_*NDM*_, *bla*_*KPC*_, *bla*_*OXA–48*_, and *bla*_*VIM*_ genes across different countries rather than a single clearly predominant carbapenemase family. These regional patterns broadly reflect the heterogeneous epidemiology of carbapenemase-producing Enterobacteriaceae reported in human, animal, and environmental settings worldwide. The repeated identification of clinically important carbapenemase-encoding genes in wildlife populations suggests possible environmental spillover originating from anthropogenic sources such as hospital wastewater, sewage contamination, agricultural runoff, and urban environmental exposure. The widespread detection of *bla*_*NDM*_ genes is particularly concerning because NDM-producing Enterobacteriaceae have been strongly associated with rapid international dissemination and plasmid-mediated transmission. Similarly, *bla*_*KPC*_ and *bla*_*OXA–48*_ genes are among the most clinically important carbapenemase-encoding genes globally and have been repeatedly associated with multidrug-resistant hospital-associated outbreaks (Alvisi et al., 2025; Halder et al., 2024; Poirel et al., 2012; Walker et al., 2025; Zhang et al., 2023).

Sequence type analysis demonstrated considerable genetic diversity among wildlife-associated CRE isolates (Figure 5). Several *E. coli* lineages were detected across different geographic regions, with ST38, ST410, and ST744 representing the most recurrent sequence types in the observed dataset. ST38 was identified in Spain, Algeria, Turkey, and USA/Alaska, whereas ST410 was detected in Spain and USA/Alaska. ST744 was reported in Spain and Chile. These lineages have previously been associated with extended-spectrum β-lactamase production, carbapenem resistance, and international dissemination in clinical, animal, and environmental settings (de Carvalho et al., 2020; Silva et al., 2025). The repeated identification of similar sequence types across geographically distant wildlife populations may therefore indicate the circulation of globally disseminated resistant lineages, although most sequence types in this study appeared to be country- or study-specific. In addition to *E. coli*, several *Klebsiella pneumoniae*, *Klebsiella quasipneumoniae*, and *Citrobacter freundii* sequence types were identified, particularly in Asia and Europe. In China, diverse *K. pneumoniae* lineages were reported, including ST1558, ST4007, ST1319, ST2541, ST2729, ST894, ST309, and ST2671, while *K. quasipneumoniae* lineages included ST35, ST1697, ST2582, ST2583, and ST2584. Spain also showed substantial MLST diversity, including *E. coli* ST38, ST410, ST744, ST1011, and ST354, along with *K. pneumoniae* ST11, ST15, ST307, ST392, and ST512. The detection of internationally relevant lineages such as *K. pneumoniae* ST11 and ST307, together with diverse carbapenemase-encoding genes among different bacterial backgrounds, is consistent with possible clonal dissemination and horizontal gene transfer among CRE lineages reported from wildlife-associated environments (Ahlstrom et al., 2023; Chudejova et al., 2024; Piispa et al., 2025; Roer et al., 2018). Together, these findings indicate that wildlife-associated samples can contain genetically diverse CRE lineages and clinically important resistance determinants, although the present data do not establish wildlife as active drivers of CRE maintenance or dissemination. The observed distribution patterns highlight that wildlife-associated CRE do not follow a single global pattern; rather, they reflect region-specific combinations of carbapenemase-encoding genes, bacterial species, and MLST lineages shaped by local environmental, anthropogenic, and epidemiological pressures.

Publication bias assessment for the overall CRE-positive proportion analysis showed asymmetry in the funnel plot (Figure 6), suggesting the possible presence of small-study effects or selective publication. However, Egger’s regression test did not provide statistically significant evidence of funnel plot asymmetry (*t* = 1.6924, *p* = 0.1088). The trim-and-fill analysis estimated four potentially missing studies on the left side of the funnel plot and reduced the adjusted pooled CRE-positive proportion from 74.49% to 59.64% (Duval and Tweedie, 2000). Nevertheless, substantial heterogeneity persisted after adjustment (*I*^2^ = 95.52%), indicating that publication bias or missing studies did not fully explain the between-study variability. Therefore, these findings should be interpreted cautiously because funnel plot asymmetry may also reflect methodological and ecological heterogeneity (Debray et al., 2019).

To facilitate appropriate interpretation of the findings and support future research, the following limitations of the present study should be considered. Because the meta-analysis was based on recovered Enterobacteriaceae isolates, the pooled estimate should not be interpreted as an estimate of wildlife-level CRE carriage prevalence or as the proportion of wild animals carrying CRE. Although the total number of wildlife samples was generally reported, the included studies did not consistently report the corresponding number of unique wild animals or wildlife samples positive for CRE. Moreover, Enterobacteriaceae-positive sample counts do not necessarily represent CRE-positive hosts, and some studies recovered multiple Enterobacteriaceae isolates from individual animals or samples. Consequently, the number of carbapenem-resistant isolates could not be reliably converted into a host-level CRE-positive numerator without introducing unsupported assumptions. Accordingly, the pooled CRE-positive proportion should not be used as a proxy for wildlife population-level CRE burden or as a direct measure of transmission risk. The number of eligible studies was relatively limited, particularly for *Citrobacter* spp. and *Enterobacter* spp., which may have reduced the robustness of some subgroup analyses. Importantly, the use of selective or enrichment culture methods in some included studies may have preferentially recovered resistant Enterobacteriaceae, thereby influencing the observed CRE-positive proportion. Consequently, the pooled estimate reflects carbapenem resistance among Enterobacteriaceae recovered under the laboratory selection procedures of the included studies. In subgroup analyses and univariate meta-regression, sample type showed the clearest association with between-study variability, whereas continent accounted for a smaller, statistically non-significant proportion of heterogeneity. Substantial residual heterogeneity remained, indicating that unmeasured ecological, methodological, and anthropogenic factors may have contributed to the observed variability. In addition, many wildlife samples originated from urban, wastewater-associated, or other anthropogenically influenced environments, making it challenging to fully differentiate wildlife-associated circulation from environmental contamination linked to human activities. Furthermore, wildlife sampled from captive, rehabilitation, rescue, or human-associated settings may differ from free-ranging wildlife in exposure to anthropogenic AMR sources, and a sensitivity analysis restricted to free-ranging wildlife was not feasible because isolate-level CRE data could not be consistently stratified according to wildlife sampling setting. Finally, the possibility of publication bias and small-study effects cannot be excluded and may have influenced the pooled CRE-positive proportion estimates.

Despite these limitations, this study provides evidence that carbapenem-resistant Enterobacteriaceae and clinically relevant resistance determinants are detectable in wildlife-associated samples across diverse ecological settings. However, the present review does not directly assess transmission dynamics, directionality of spread, or reservoir status. Therefore, wildlife should not be interpreted as confirmed primary reservoirs or active drivers of CRE dissemination based on the current evidence. Instead, wildlife may serve as potential sentinels or incidental carriers reflecting environmental contamination, particularly in aquatic, wastewater-associated, or anthropogenically influenced ecosystems. Future wildlife AMR surveillance studies should provide detailed descriptions of culture and enrichment procedures and clearly report the total number of individual animals or samples screened, the number positive for CRE, and the number of Enterobacteriaceae isolates recovered and tested. Where feasible, reporting results from non-selective alongside selective isolation approaches would further facilitate distinction between host-level CRE carriage and resistance patterns among selectively recovered bacterial isolates. Further longitudinal surveillance, ecological sampling, and genomic transmission studies are needed to clarify the role of wildlife in CRE exposure, carriage, and possible transmission pathways within One Health networks.

## Data Availability

The original contributions presented in this study are included in this article/Supplementary material, further inquiries can be directed to the corresponding author.

## Supplementary material

Supplementary Table 1 Preferred Reporting Items for Systematic Reviews and Meta-Analyses (PRISMA) 2020 checklist.
