# Supplementary material for: Occurrence and molecular characteristics of carbapenem-resistant Enterobacteriaceae recovered from wildlife: a systematic review and meta-analysis
Source: Front Microbiol. 2026 Oct 16;17:1897815. doi: 10.3389/fmicb.2026.1897815 (PMC13719466; doi:10.3389/fmicb.2026.1897815)

Supplementary Figures

Figure S1. Sensitivity analysis of CRE-positive proportion among recovered Enterobacteriaceae isolates. The CRE-positive proportion estimates (right) and their corresponding 95% confidence intervals were computed by leaving out each study (left).

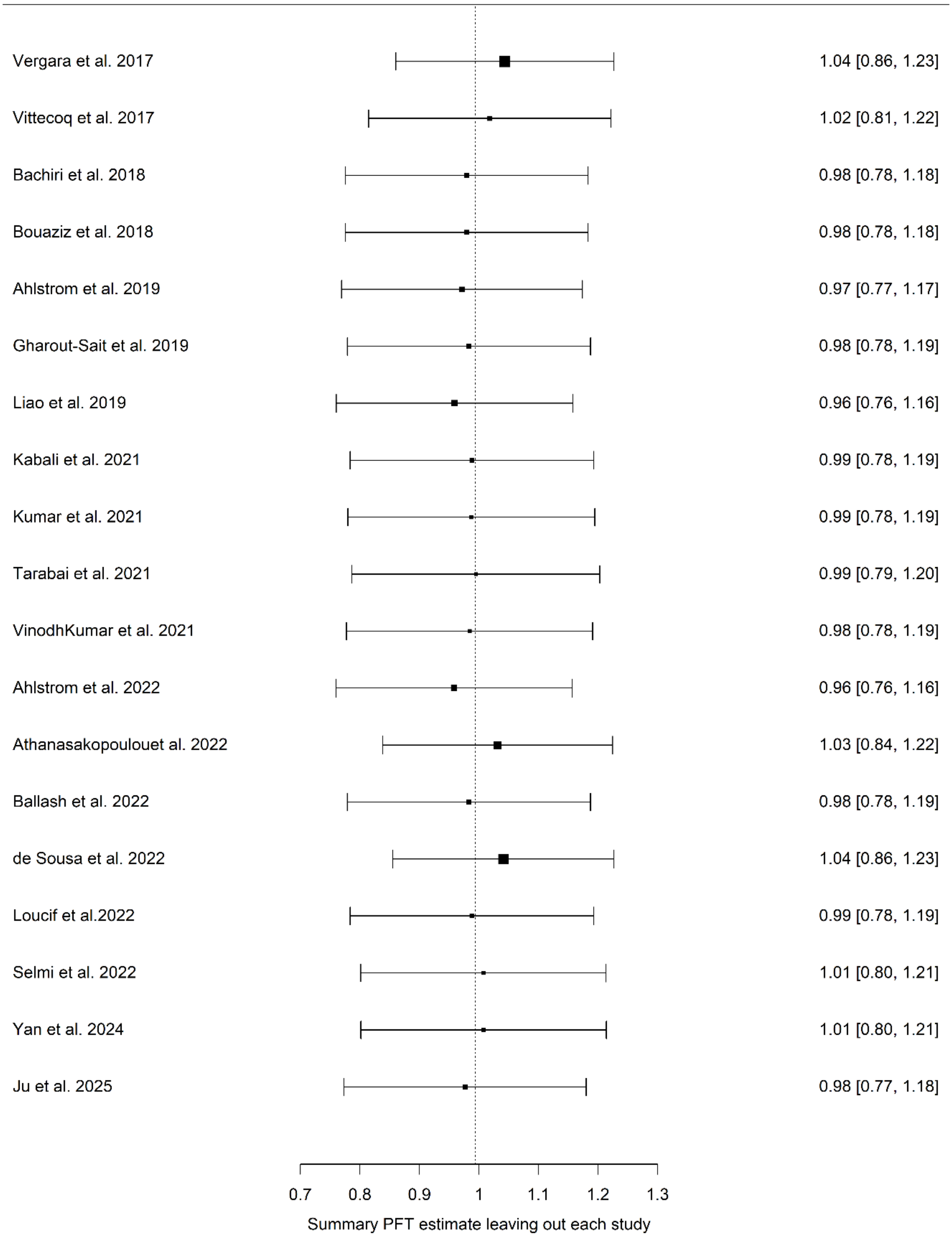

Figure S2. Subgroup analysis of CRE-positive proportion based on Enterobacteriaceae species group.

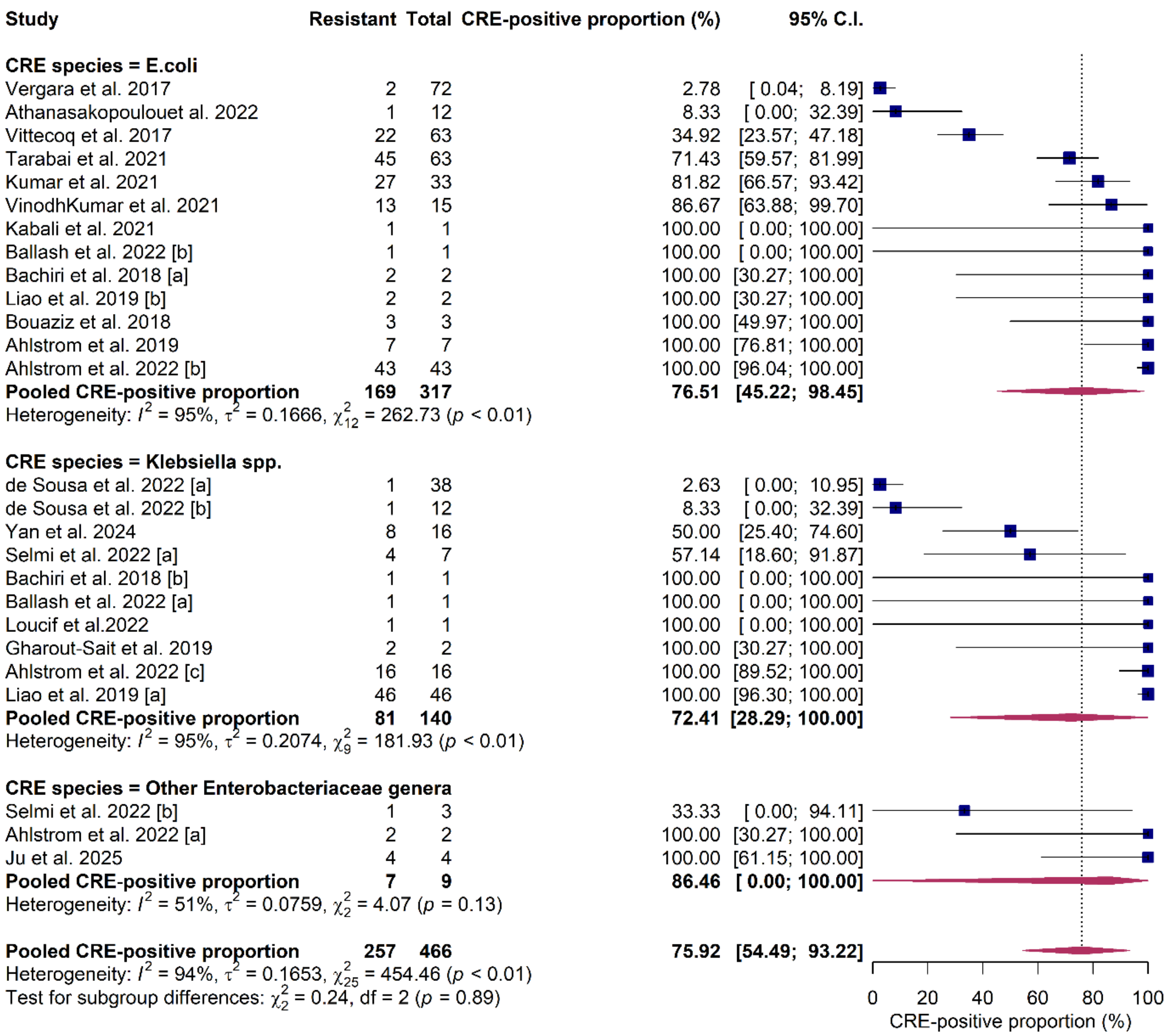

Figure S3. Subgroup analysis of CRE-positive proportion based on wildlife taxonomic order.

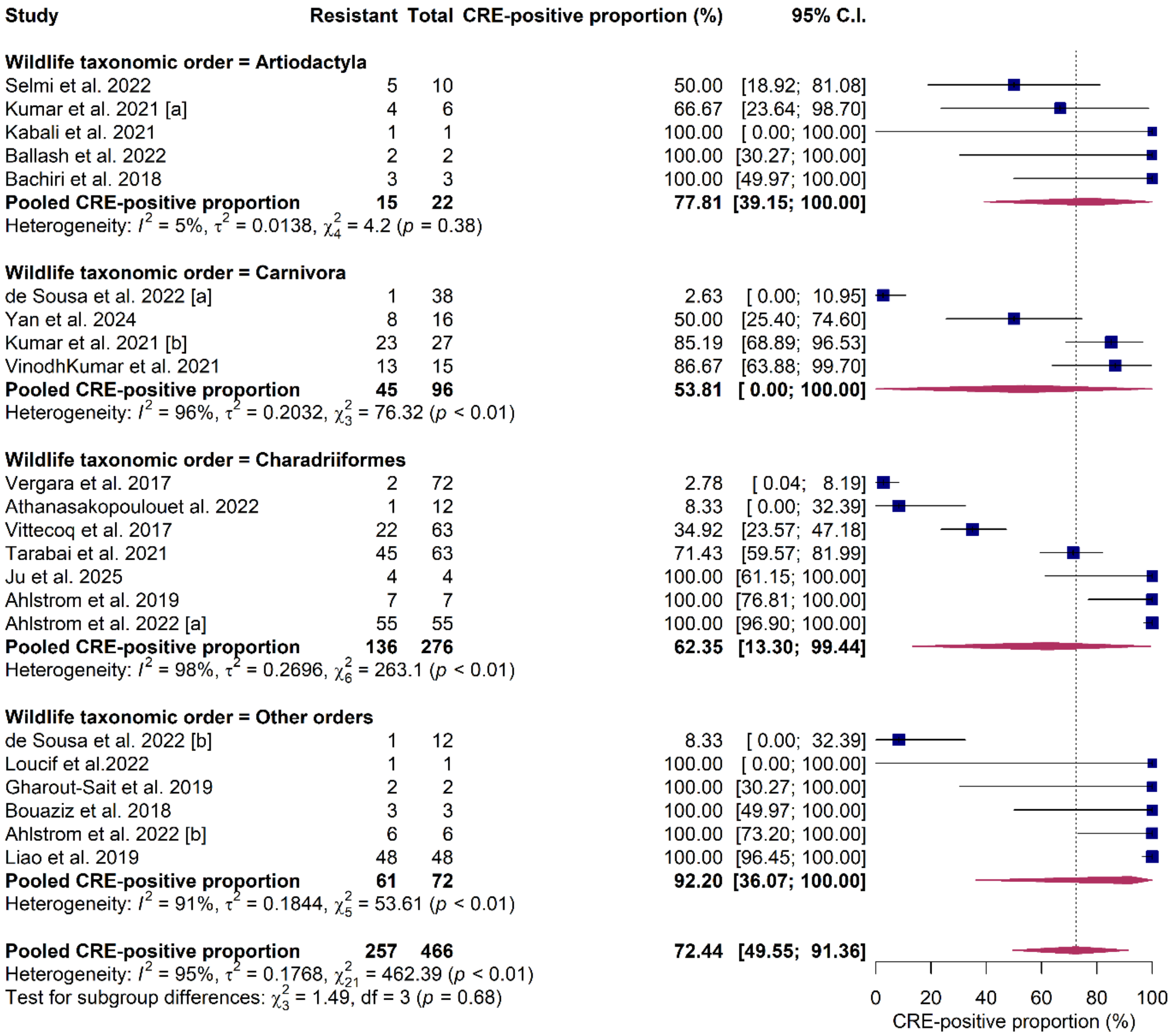

Figure S4. Subgroup analysis of CRE-positive proportion based on carbapenem agent.

Study	Resistant	Total	CRE-positive proportion (%)	95% C.I.
**Carbapenem agent = Ertapenem**				
Selmi et al. 2022	5	10	50.00	[18.92; 81.08]
Kumar et al. 2021 [b]	7	11	63.64	[32.69; 90.00]
Tarabai et al. 2021 [b]	15	21	71.43	[49.96; 89.08]
VinodhKumar et al. 2021 [b]	4	5	80.00	[32.49; 100.00]
Loucif et al.2022	1	1	100.00	[0.00; 100.00]
Gharout-Sait et al. 2019	2	2	100.00	[30.27; 100.00]
Bachiri et al. 2018 [a]	3	3	100.00	[49.97; 100.00]
Bouaziz et al. 2018 [a]	3	3	100.00	[49.97; 100.00]
Ju et al. 2025 [b]	4	4	100.00	[61.15; 100.00]
Liao et al. 2019 [b]	48	48	100.00	[96.45; 100.00]
**Pooled CRE-positive proportion**	**92**	**108**	**90.81**	**[72.50; 100.00]**
Heterogeneity: $I^2 = 73\%$, $\tau^2 = 0.0461$, $\chi^2_9 = 32.8$ ($p < 0.01$)				
**Carbapenem agent = Imipenem**				
de Sousa et al. 2022 [a]	2	25	8.00	[0.15; 22.62]
Athanasakopoulouet al. 2022	1	12	8.33	[0.00; 32.39]
Vittecoq et al. 2017	22	63	34.92	[23.57; 47.18]
Tarabai et al. 2021 [a]	15	21	71.43	[49.96; 89.08]
VinodhKumar et al. 2021 [a]	4	5	80.00	[32.49; 100.00]
Kumar et al. 2021 [a]	9	11	81.82	[52.65; 99.53]
Yan et al. 2024 [b]	7	8	87.50	[53.79; 100.00]
Kabali et al. 2021	1	1	100.00	[0.00; 100.00]
Ju et al. 2025 [a]	4	4	100.00	[61.15; 100.00]
Liao et al. 2019 [a]	48	48	100.00	[96.45; 100.00]
**Pooled CRE-positive proportion**	**113**	**198**	**67.73**	**[33.55; 94.98]**
Heterogeneity: $I^2 = 94\%$, $\tau^2 = 0.1552$, $\chi^2_9 = 147.41$ ($p < 0.01$)				
**Carbapenem agent = Meropenem**				
de Sousa et al. 2022 [b]	0	25	0.00	[0.00; 6.77]
Vergara et al. 2017	2	72	2.78	[0.04; 8.19]
Yan et al. 2024 [a]	1	8	12.50	[0.00; 46.21]
Tarabai et al. 2021 [c]	15	21	71.43	[49.96; 89.08]
Ballash et al. 2022	2	2	100.00	[30.27; 100.00]
Bachiri et al. 2018 [b]	3	3	100.00	[49.97; 100.00]
Bouaziz et al. 2018 [b]	3	3	100.00	[49.97; 100.00]
Ju et al. 2025 [c]	4	4	100.00	[61.15; 100.00]
VinodhKumar et al. 2021 [c]	5	5	100.00	[68.27; 100.00]
Ahlstrom et al. 2019	7	7	100.00	[76.81; 100.00]
Kumar et al. 2021 [c]	11	11	100.00	[84.93; 100.00]
Liao et al. 2019 [c]	48	48	100.00	[96.45; 100.00]
Ahlstrom et al. 2022	61	61	100.00	[97.20; 100.00]
**Pooled CRE-positive proportion**	**162**	**270**	**80.69**	**[46.26; 100.00]**
Heterogeneity: $I^2 = 97\%$, $\tau^2 = 0.2571$, $\chi^2_{12} = 415.28$ ($p < 0.01$)				
**Pooled CRE-positive proportion**	**367**	**576**	**79.83**	**[63.47; 93.02]**
Heterogeneity: $I^2 = 95\%$, $\tau^2 = 0.1628$, $\chi^2_{32} = 630.56$ ($p < 0.01$)				
Test for subgroup differences: $\chi^2_2 = 1.90$, df = 2 ($p = 0.39$)				

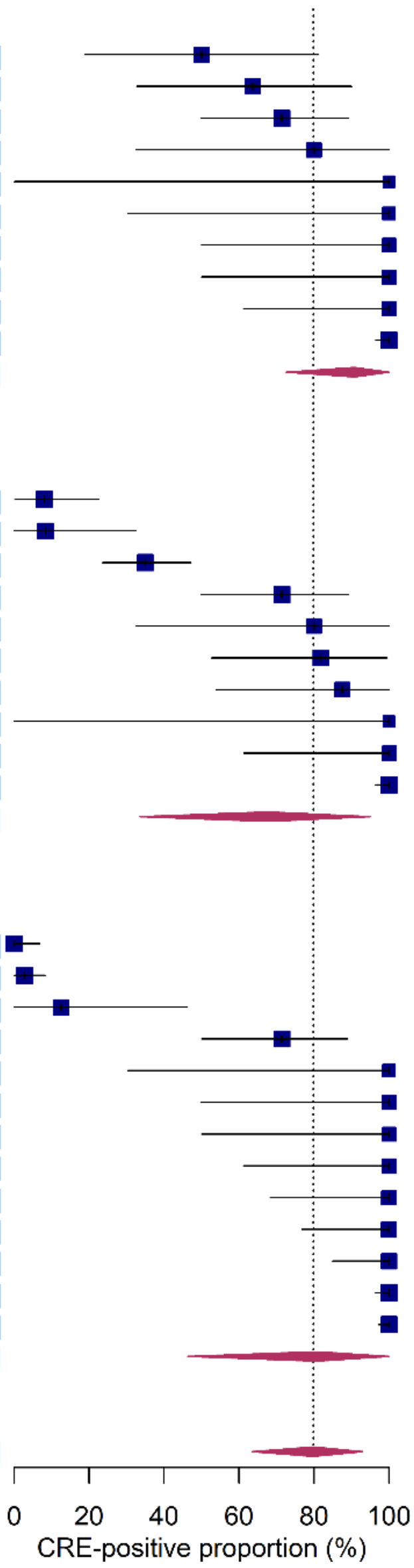

Figure S5. Subgroup analysis of CRE-positive proportion based on susceptibility testing method.

Study	Resistant	Total	CRE-positive proportion (%)	95% C.I.
**Susceptibility testing = Disk diffusion test**				
de Sousa et al. 2022	2	50	4.00	[0.07; 11.68]
Athanasakopoulouet al. 2022	1	12	8.33	[0.00; 32.39]
Vittecoq et al. 2017	22	63	34.92	[23.57; 47.18]
Selmi et al. 2022	5	10	50.00	[18.92; 81.08]
Yan et al. 2024	8	16	50.00	[25.40; 74.60]
Tarabai et al. 2021	45	63	71.43	[59.57; 81.99]
Kumar et al. 2021	27	33	81.82	[66.57; 93.42]
VinodhKumar et al. 2021	13	15	86.67	[63.88; 99.70]
Kabali et al. 2021	1	1	100.00	[0.00; 100.00]
Loucif et al.2022	1	1	100.00	[0.00; 100.00]
Gharout-Sait et al. 2019	2	2	100.00	[30.27; 100.00]
Bachiri et al. 2018	3	3	100.00	[49.97; 100.00]
Bouaziz et al. 2018	3	3	100.00	[49.97; 100.00]
Ahlstrom et al. 2022	61	61	100.00	[97.20; 100.00]
**Pooled CRE-positive proportion**	**194**	**333**	**69.62**	**[41.94; 92.42]**
Heterogeneity: I^2 = 95%, τ^2 = 0.1392, χ^2_{13} = 238.76 (p < 0.01)				
**Susceptibility testing = MIC test**				
Vergara et al. 2017	2	72	2.78	[0.04; 8.19]
Ballash et al. 2022	2	2	100.00	[30.27; 100.00]
Ju et al. 2025	4	4	100.00	[61.15; 100.00]
Ahlstrom et al. 2019	7	7	100.00	[76.81; 100.00]
Liao et al. 2019	48	48	100.00	[96.45; 100.00]
**Pooled CRE-positive proportion**	**63**	**133**	**85.75**	**[11.33; 100.00]**
Heterogeneity: I^2 = 98%, τ^2 = 0.3075, χ^2_4 = 222.04 (p < 0.01)				
**Pooled CRE-positive proportion**	**257**	**466**	**74.49**	**[49.75; 94.04]**
Heterogeneity: I^2 = 96%, τ^2 = 0.1757, χ^2_{18} = 466.03 (p < 0.01)				
Test for subgroup differences: χ^2_1 = 0.38, df = 1 (p = 0.54)				

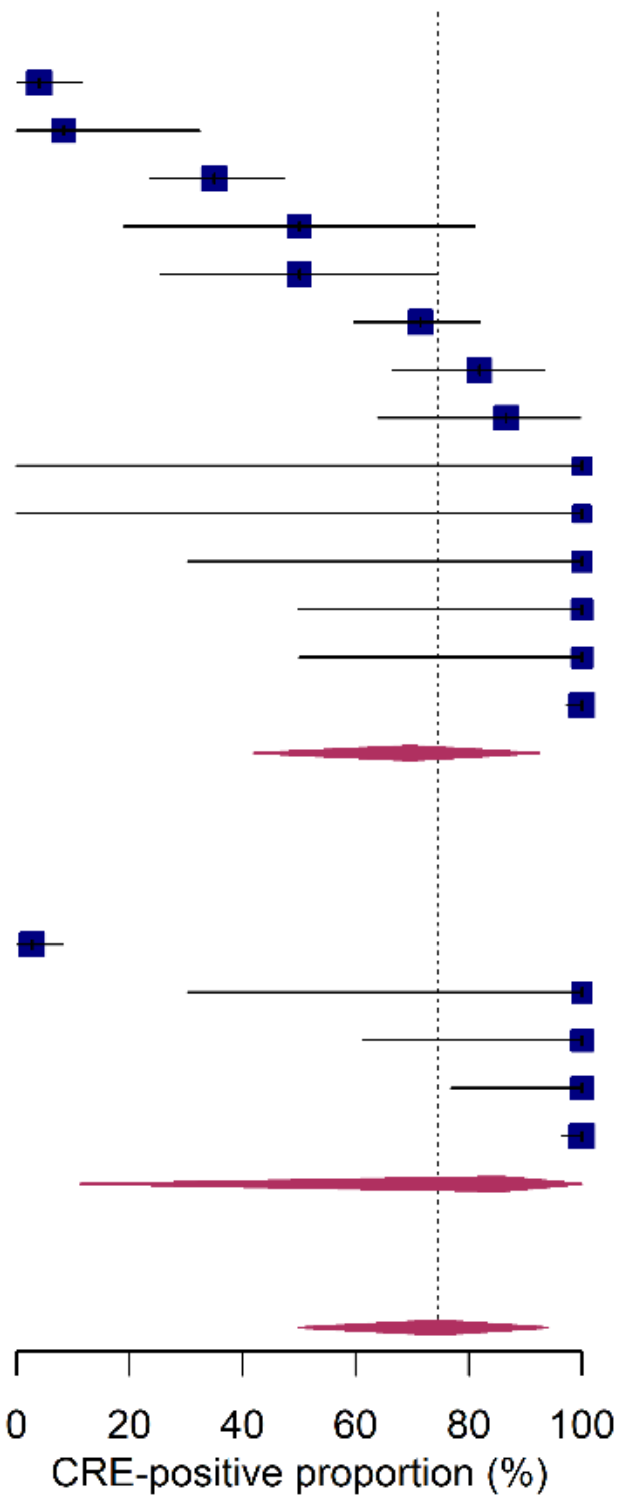

Figure S6. Subgroup analysis of CRE-positive proportion based on sample type.

Study	Resistant	Total	CRE-positive proportion (%)	95% C.I.
**Sample type = Fecal samples**				
Vergara et al. 2017	2	72	2.78	[0.04; 8.19]
Selmi et al. 2022	5	10	50.00	[18.92; 81.08]
Yan et al. 2024	8	16	50.00	[25.40; 74.60]
Kumar et al. 2021	27	33	81.82	[66.57; 93.42]
VinodhKumar et al. 2021	13	15	86.67	[63.88; 99.70]
Kabali et al. 2021	1	1	100.00	[0.00; 100.00]
Loucif et al.2022	1	1	100.00	[0.00; 100.00]
Gharout-Sait et al. 2019	2	2	100.00	[30.27; 100.00]
Ballash et al. 2022	2	2	100.00	[30.27; 100.00]
Bachiri et al. 2018	3	3	100.00	[49.97; 100.00]
Bouaziz et al. 2018	3	3	100.00	[49.97; 100.00]
Ju et al. 2025	4	4	100.00	[61.15; 100.00]
Ahlstrom et al. 2019	7	7	100.00	[76.81; 100.00]
Liao et al. 2019	48	48	100.00	[96.45; 100.00]
Ahlstrom et al. 2022	61	61	100.00	[97.20; 100.00]
**Pooled CRE-positive proportion**	**187**	**278**	**89.29**	**[66.91; 100.00]**
Heterogeneity: I^2 = 96%, τ^2 = 0.1326, χ^2_{14} = 323.33 (p < 0.01)				
**Sample type = Rectal/cloacal/wound swab**				
de Sousa et al. 2022 [a]	1	38	2.63	[0.00; 10.95]
Athanasakopoulouet al. 2022	1	12	8.33	[0.00; 32.39]
de Sousa et al. 2022 [b]	1	12	8.33	[0.00; 32.39]
Vittecoq et al. 2017	22	63	34.92	[23.57; 47.18]
Tarabai et al. 2021	45	63	71.43	[59.57; 81.99]
**Pooled CRE-positive proportion**	**70**	**188**	**23.04**	**[0.00; 63.97]**
Heterogeneity: I^2 = 95%, τ^2 = 0.1011, χ^2_4 = 72.86 (p < 0.01)				
**Pooled CRE-positive proportion**	**257**	**466**	**70.48**	**[45.39; 91.33]**
Heterogeneity: I^2 = 96%, τ^2 = 0.1868, χ^2_{19} = 465.44 (p < 0.01)				
Test for subgroup differences: χ^2_1 = 12.28, df = 1 (p < 0.01)				

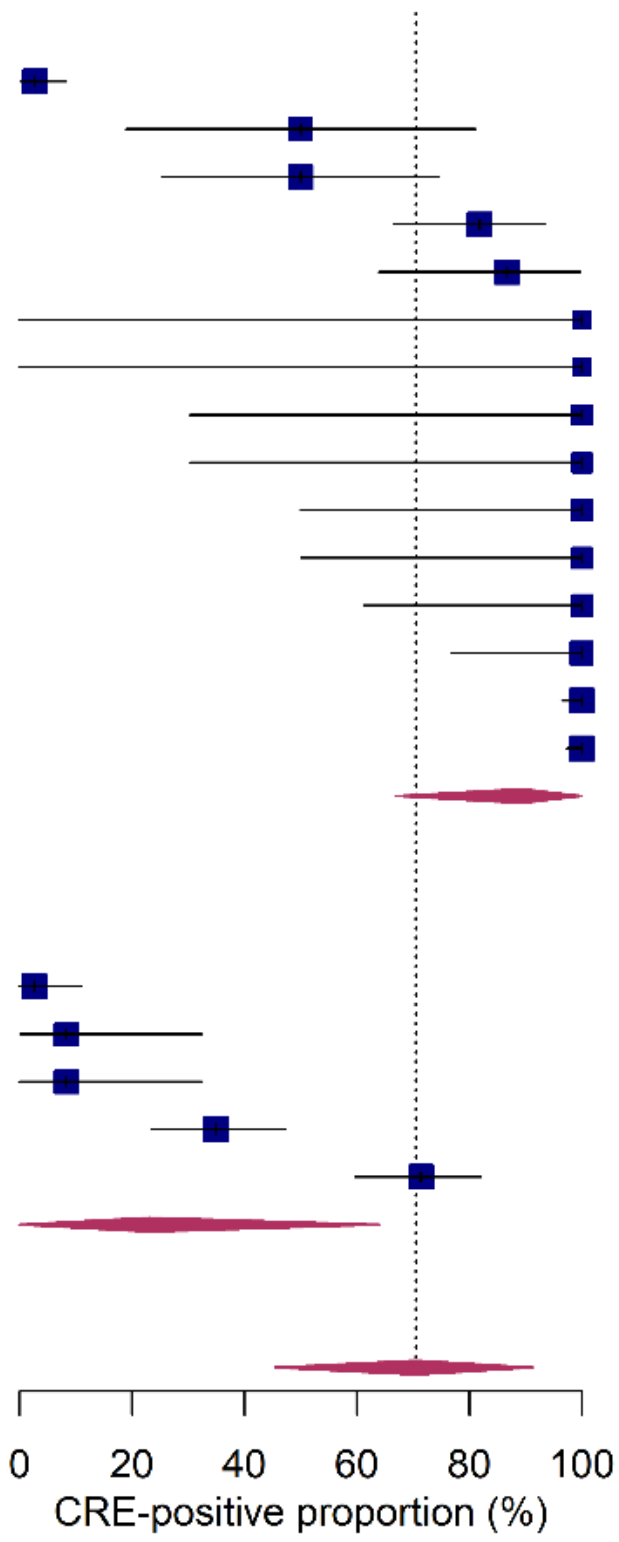

Figure S7. Subgroup analysis of CRE-positive proportion based on sample size.

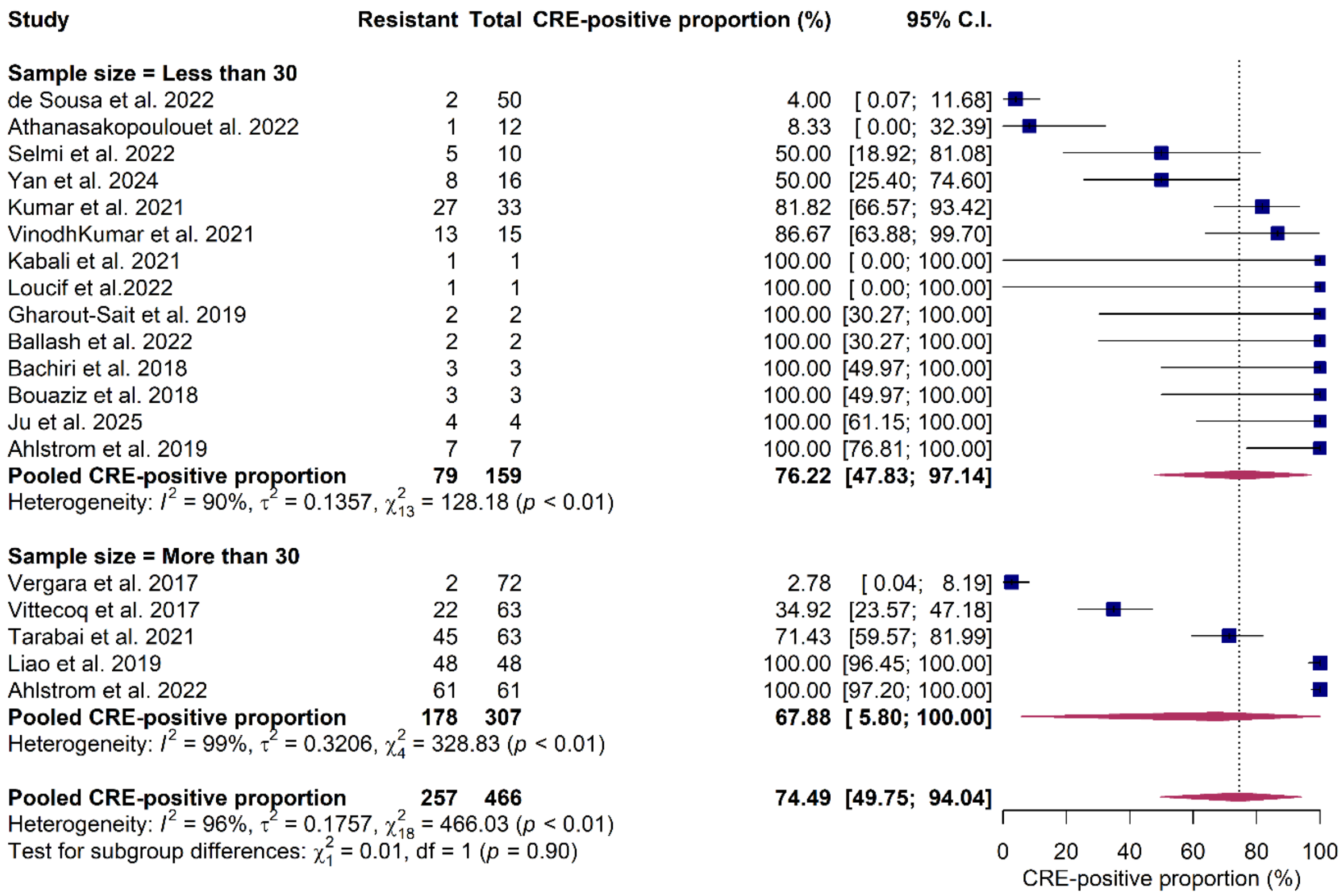

Figure S8. Subgroup analysis of CRE-positive proportion based on geographical region.

Study	Resistant	Total	CRE-positive proportion (%)	95% C.I.
**Geographical region = Africa**				
Selmi et al. 2022	5	10	50.00	[18.92; 81.08]
Kabali et al. 2021	1	1	100.00	[0.00; 100.00]
Loucif et al.2022	1	1	100.00	[0.00; 100.00]
Gharout-Sait et al. 2019	2	2	100.00	[30.27; 100.00]
Bachiri et al. 2018	3	3	100.00	[49.97; 100.00]
Bouaziz et al. 2018	3	3	100.00	[49.97; 100.00]
**Pooled CRE-positive proportion**	**15**	**20**	**91.53**	**[58.25; 100.00]**
Heterogeneity: I^2 = 9%, τ^2 = 0.0307, χ^2_5 = 5.47 (p = 0.36)				
**Geographical region = Americas**				
de Sousa et al. 2022	2	50	4.00	[0.07; 11.68]
Ballash et al. 2022	2	2	100.00	[30.27; 100.00]
Ahlstrom et al. 2019	7	7	100.00	[76.81; 100.00]
Ahlstrom et al. 2022 [b]	16	16	100.00	[89.52; 100.00]
**Pooled CRE-positive proportion**	**27**	**75**	**80.45**	**[0.00; 100.00]**
Heterogeneity: I^2 = 97%, τ^2 = 0.3447, χ^2_3 = 99.13 (p < 0.01)				
**Geographical region = Asia.Pacific**				
Yan et al. 2024	8	16	50.00	[25.40; 74.60]
Tarabai et al. 2021	45	63	71.43	[59.57; 81.99]
Kumar et al. 2021	27	33	81.82	[66.57; 93.42]
VinodhKumar et al. 2021	13	15	86.67	[63.88; 99.70]
Ju et al. 2025	4	4	100.00	[61.15; 100.00]
Ahlstrom et al. 2022 [a]	14	14	100.00	[88.07; 100.00]
Liao et al. 2019	48	48	100.00	[96.45; 100.00]
**Pooled CRE-positive proportion**	**159**	**193**	**88.33**	**[67.15; 99.90]**
Heterogeneity: I^2 = 86%, τ^2 = 0.0542, χ^2_6 = 42.87 (p < 0.01)				
**Geographical region = Europe**				
Vergara et al. 2017	2	72	2.78	[0.04; 8.19]
Athanasakopoulouet al. 2022	1	12	8.33	[0.00; 32.39]
Vittecoq et al. 2017	22	63	34.92	[23.57; 47.18]
Ahlstrom et al. 2022 [c]	31	31	100.00	[94.53; 100.00]
**Pooled CRE-positive proportion**	**56**	**178**	**37.35**	**[0.00; 100.00]**
Heterogeneity: I^2 = 98%, τ^2 = 0.3269, χ^2_3 = 151.29 (p < 0.01)				
**Pooled CRE-positive proportion**	**257**	**466**	**78.99**	**[56.68; 95.82]**
Heterogeneity: I^2 = 96%, τ^2 = 0.1750, χ^2_{20} = 454.14 (p < 0.01)				
Test for subgroup differences: χ^2_3 = 3.02, df = 3 (p = 0.39)				

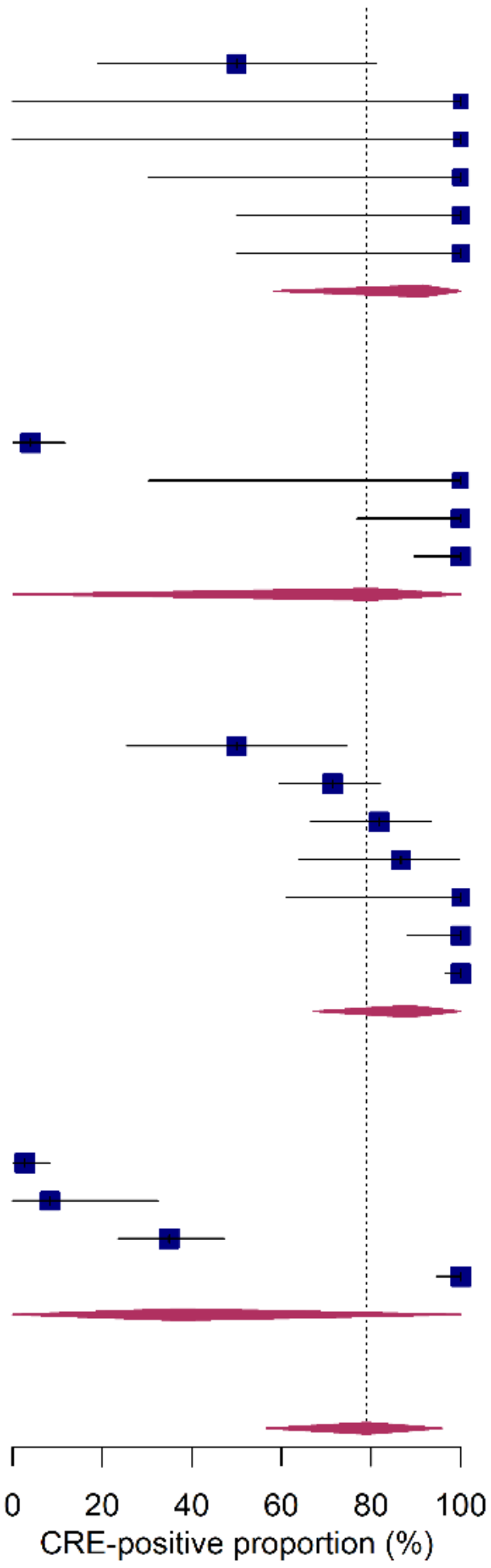

Figure S9. Subgroup analysis of CRE-positive proportion based on publication year.

Study	Resistant	Total	CRE-positive proportion (%)	95% C.I.
**Publication year = After 2020**				
de Sousa et al. 2022	2	50	4.00	[0.07; 11.68]
Athanasakopoulouet al. 2022	1	12	8.33	[0.00; 32.39]
Selmi et al. 2022	5	10	50.00	[18.92; 81.08]
Yan et al. 2024	8	16	50.00	[25.40; 74.60]
Tarabai et al. 2021	45	63	71.43	[59.57; 81.99]
Kumar et al. 2021	27	33	81.82	[66.57; 93.42]
VinodhKumar et al. 2021	13	15	86.67	[63.88; 99.70]
Kabali et al. 2021	1	1	100.00	[0.00; 100.00]
Loucif et al.2022	1	1	100.00	[0.00; 100.00]
Ballash et al. 2022	2	2	100.00	[30.27; 100.00]
Ju et al. 2025	4	4	100.00	[61.15; 100.00]
Ahlstrom et al. 2022	61	61	100.00	[97.20; 100.00]
**Pooled CRE-positive proportion**	**170**	**268**	**71.16**	**[39.74; 95.63]**
Heterogeneity: $I^2 = 95\%$, $\tau^2 = 0.1502$, $\chi^2_{11} = 216.51$ ($p < 0.01$)				
**Publication year = Before 2020**				
Vergara et al. 2017	2	72	2.78	[0.04; 8.19]
Vittecoq et al. 2017	22	63	34.92	[23.57; 47.18]
Gharout-Sait et al. 2019	2	2	100.00	[30.27; 100.00]
Bachiri et al. 2018	3	3	100.00	[49.97; 100.00]
Bouaziz et al. 2018	3	3	100.00	[49.97; 100.00]
Ahlstrom et al. 2019	7	7	100.00	[76.81; 100.00]
Liao et al. 2019	48	48	100.00	[96.45; 100.00]
**Pooled CRE-positive proportion**	**87**	**198**	**80.11**	**[27.18; 100.00]**
Heterogeneity: $I^2 = 97\%$, $\tau^2 = 0.2463$, $\chi^2_{6} = 228.35$ ($p < 0.01$)				
**Pooled CRE-positive proportion**	**257**	**466**	**74.49**	**[49.75; 94.04]**
Heterogeneity: $I^2 = 96\%$, $\tau^2 = 0.1757$, $\chi^2_{18} = 466.03$ ($p < 0.01$)				
Test for subgroup differences: $\chi^2_1 = 0.18$, df = 1 ($p = 0.68$)				

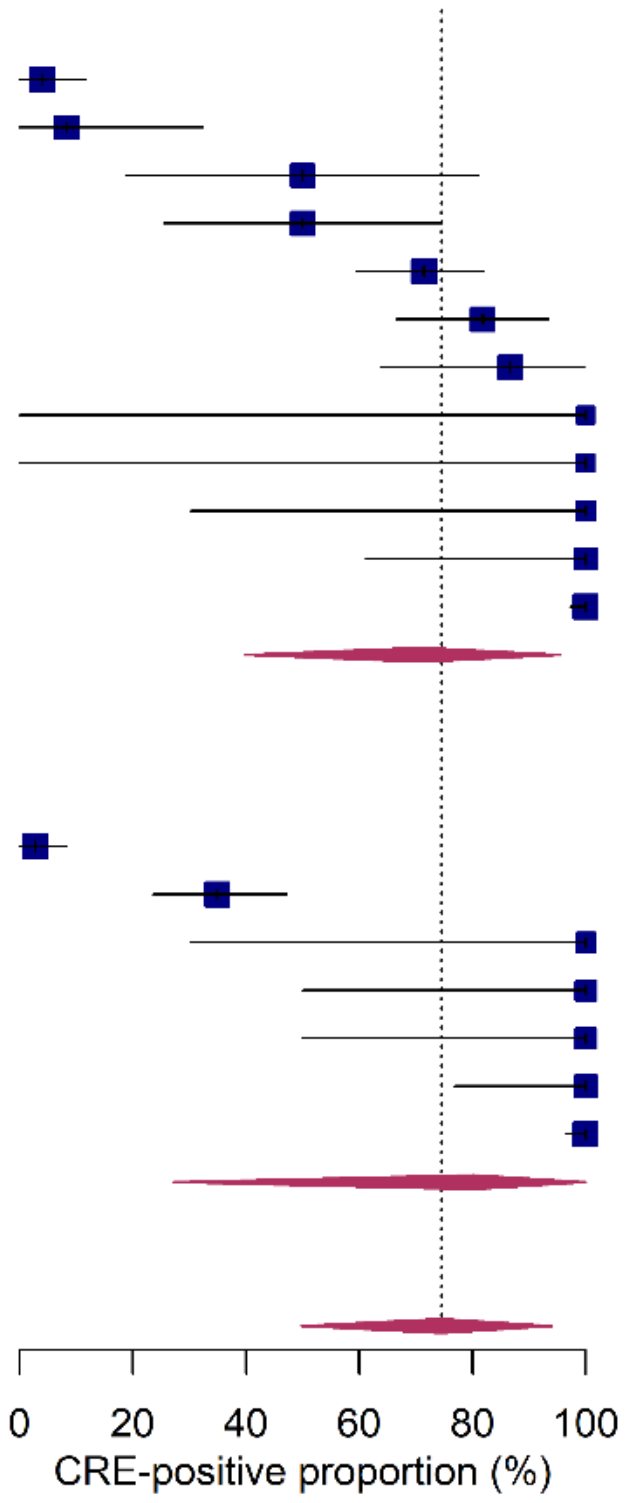

Figure S10. Subgroup analysis of CRE-positive proportion based on sampling environment.

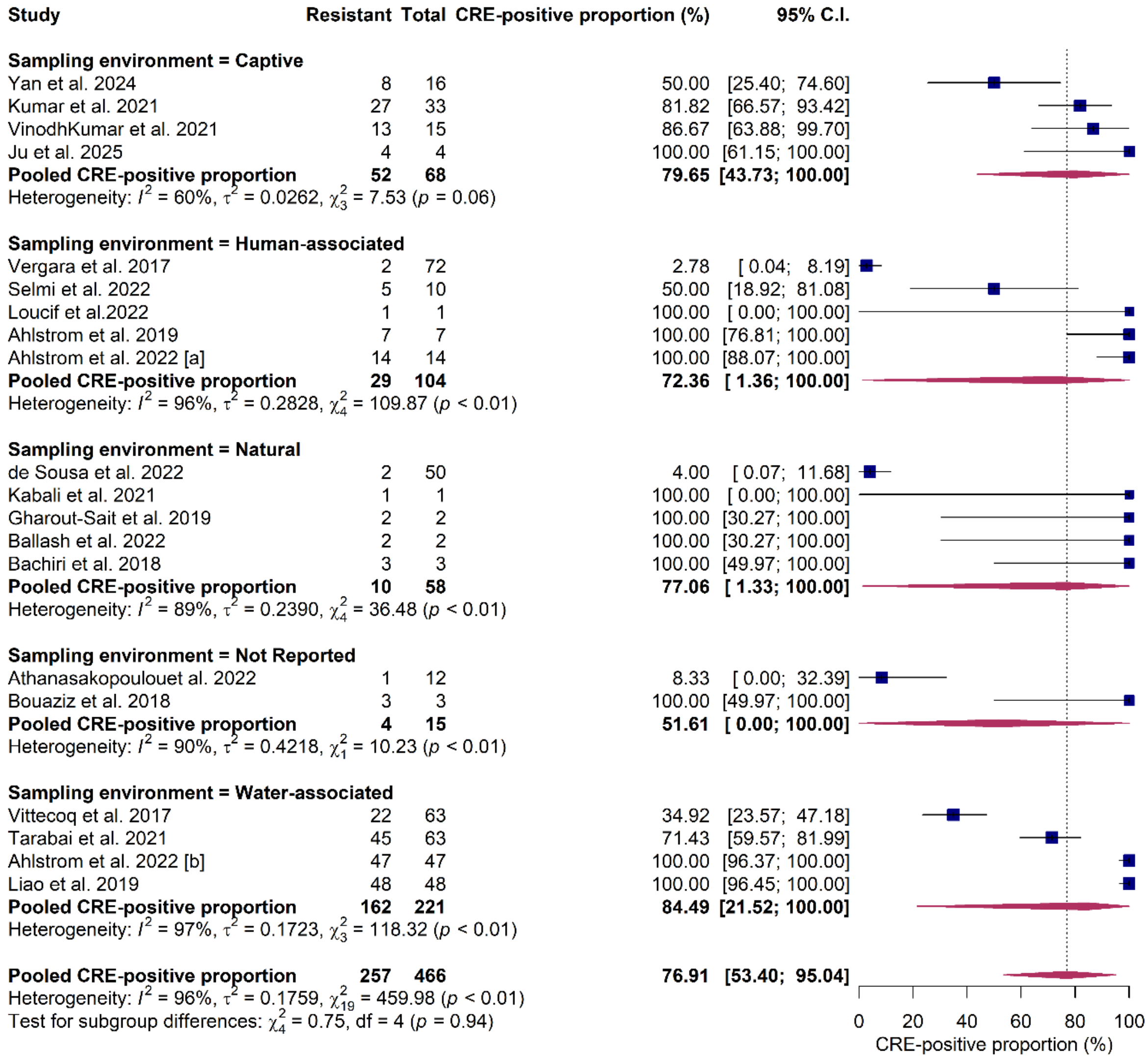

Figure S11. Univariate meta-regression of CRE-positive proportion based on Enterobacteriaceae species group.

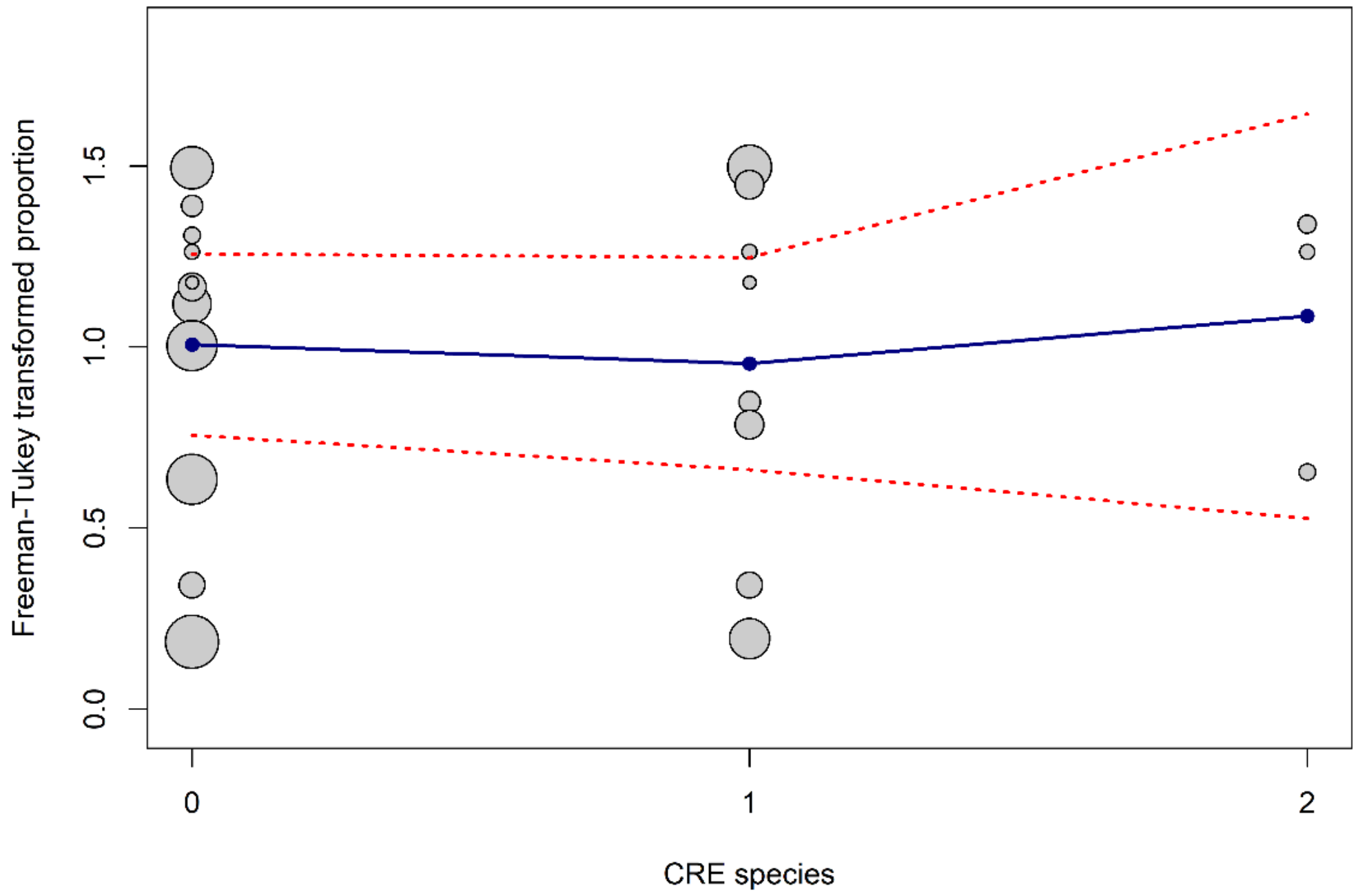

Figure S12. Univariate meta-regression of CRE-positive proportion based on wildlife taxonomic order.

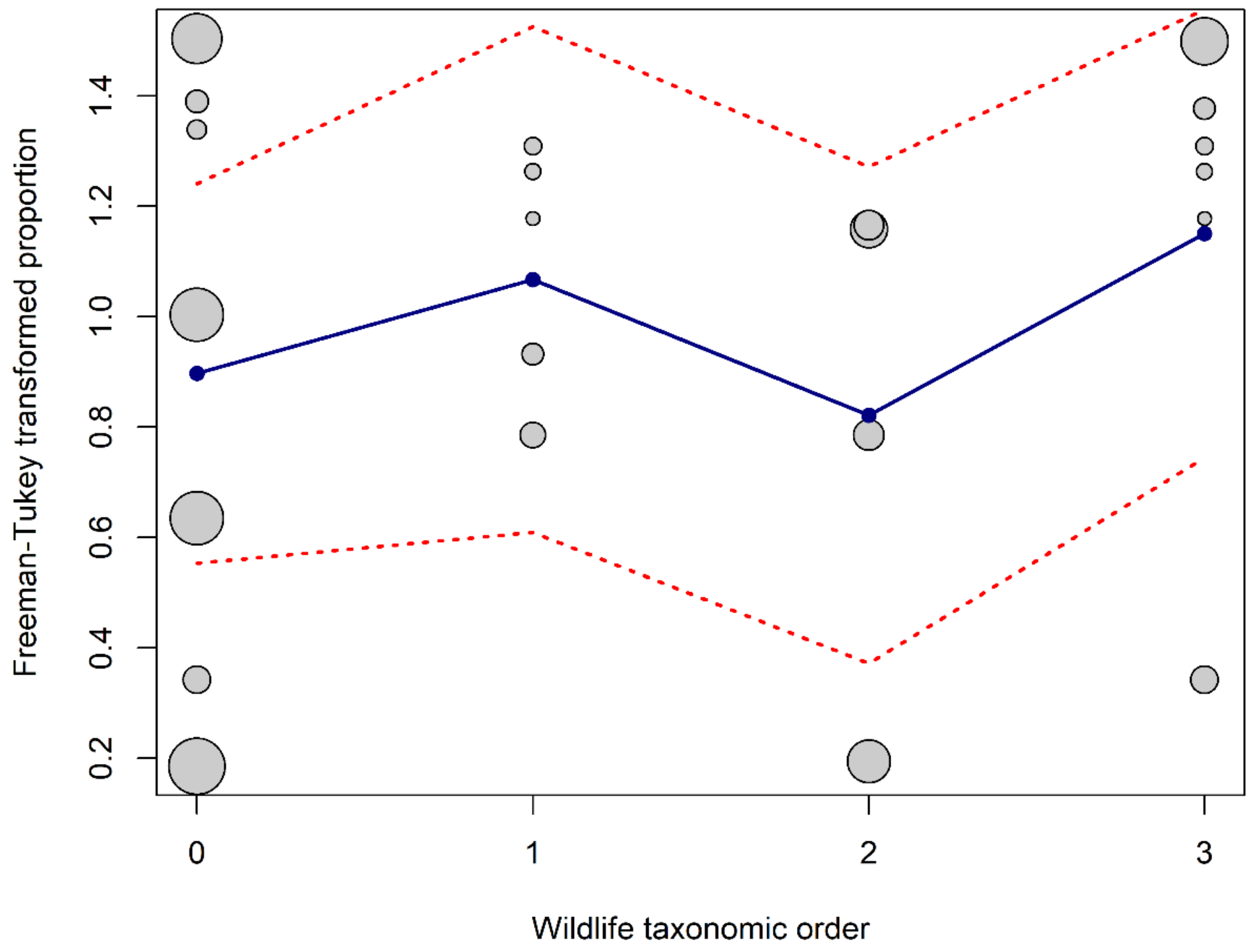

Figure S13. Univariate meta-regression of CRE-positive proportion based on carbapenem agent.

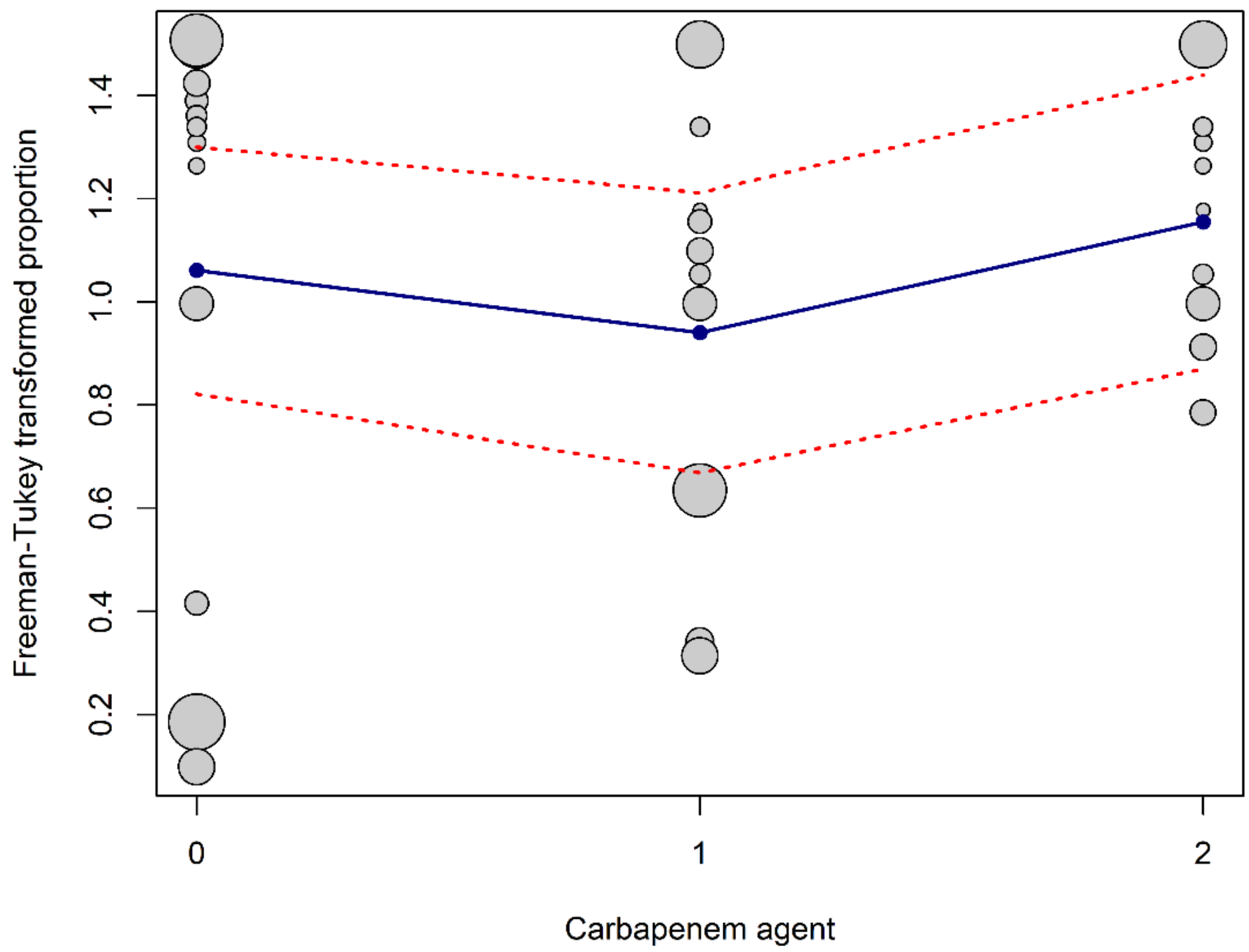

Figure S14. Univariate meta-regression of CRE-positive proportion based on susceptibility testing method.

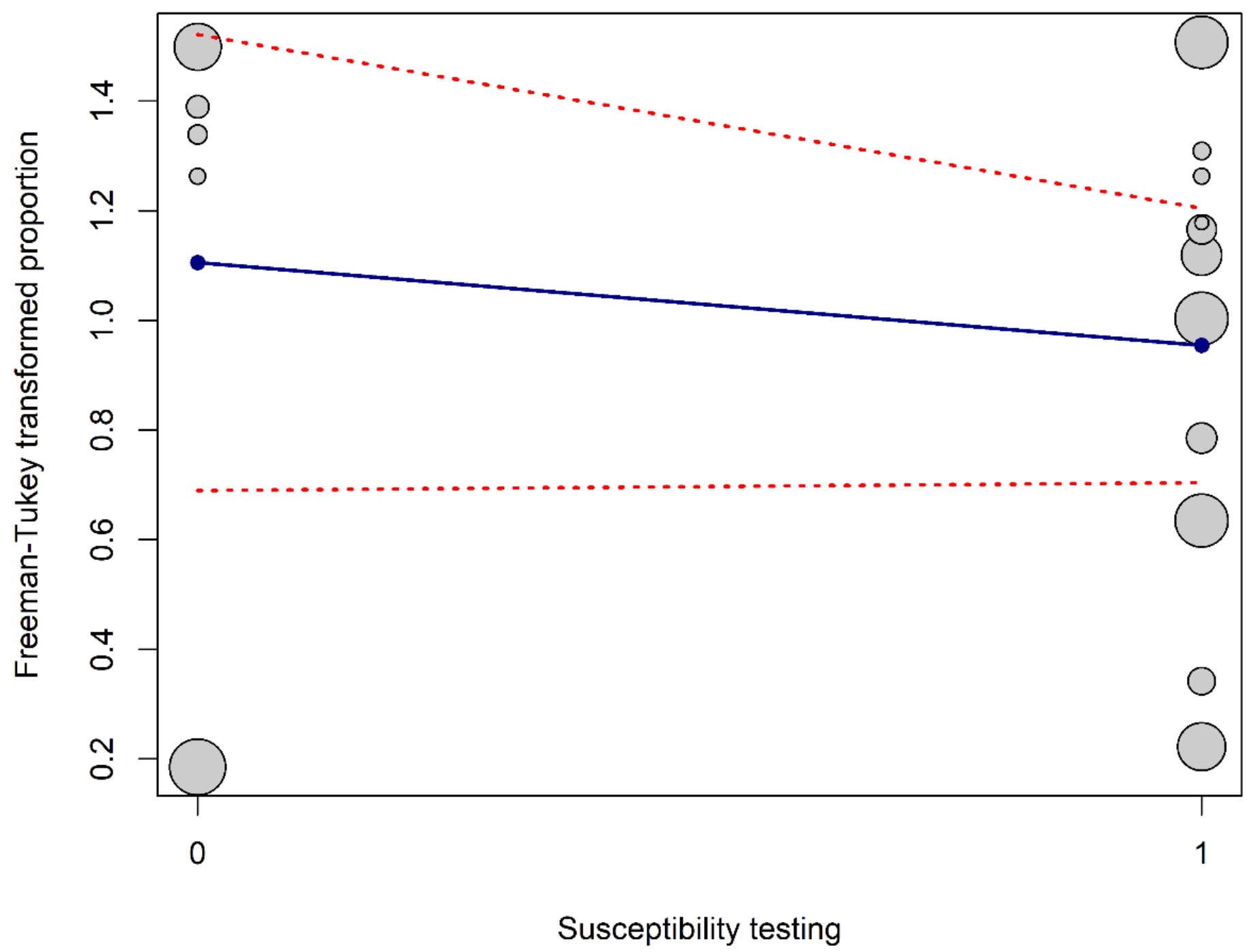

Figure S15. Univariate meta-regression of CRE-positive proportion based on sample type.

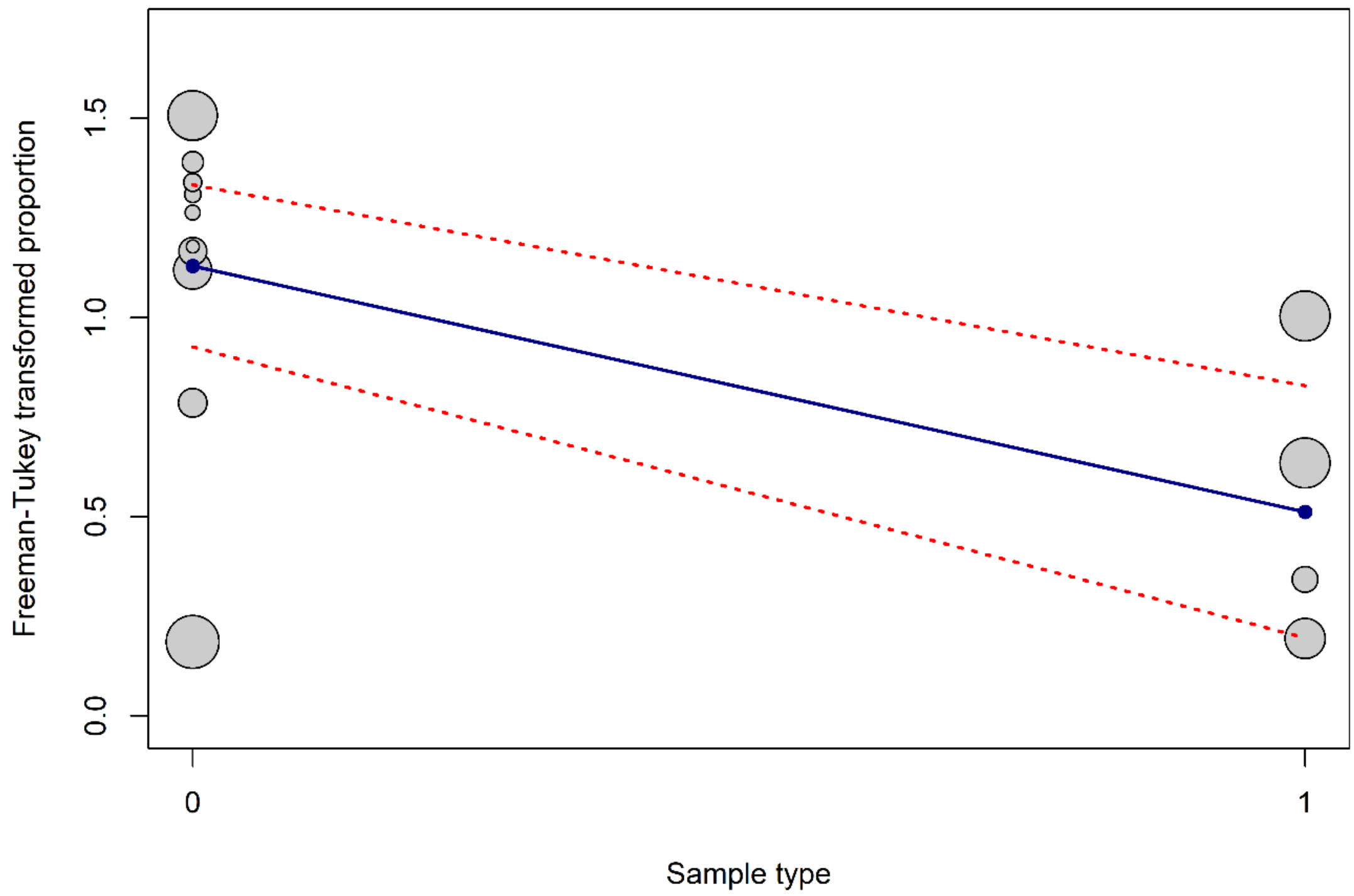

Figure S16. Univariate meta-regression of CRE-positive proportion based on sample size.

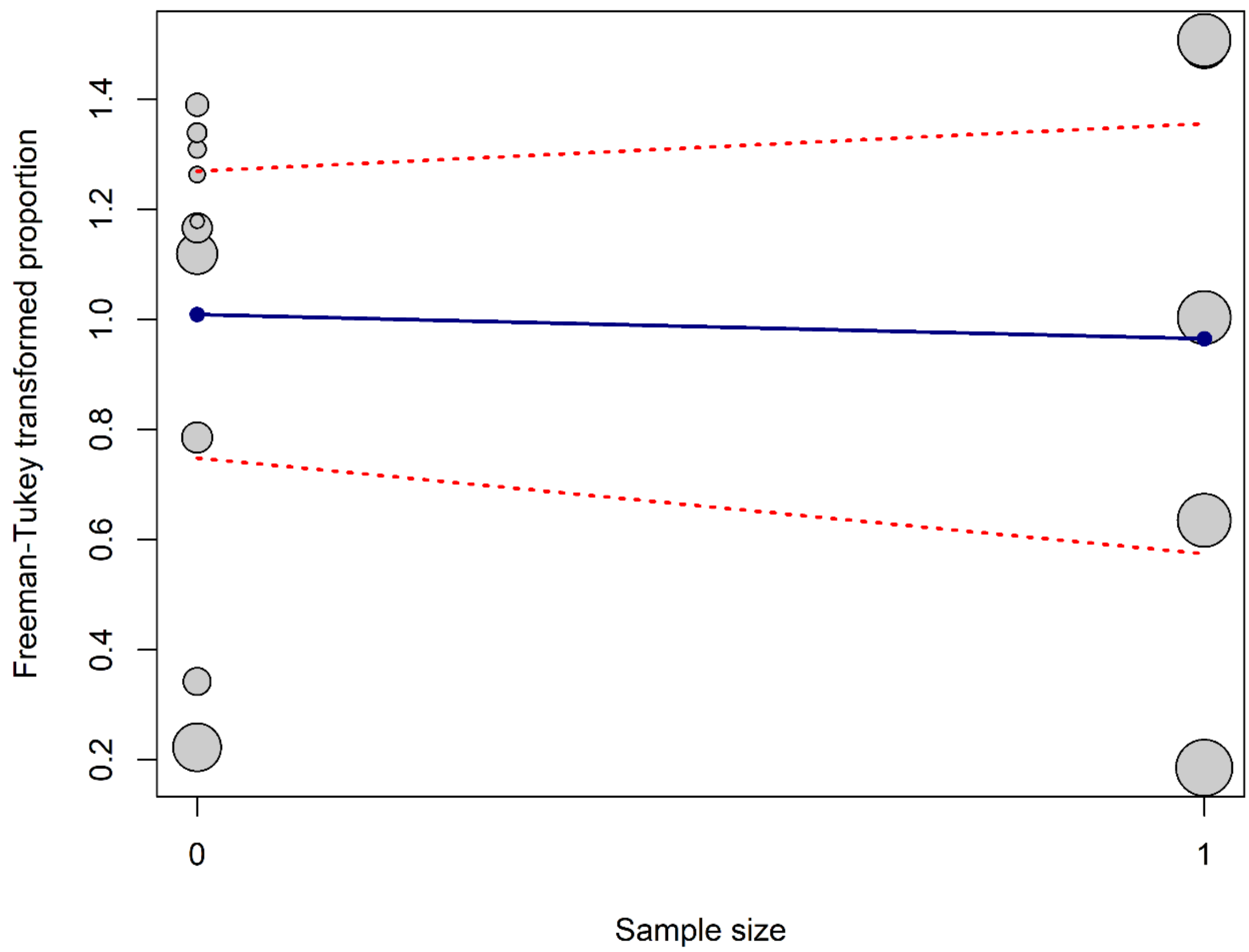

Figure S17. Univariate meta-regression of CRE-positive proportion based on continent.

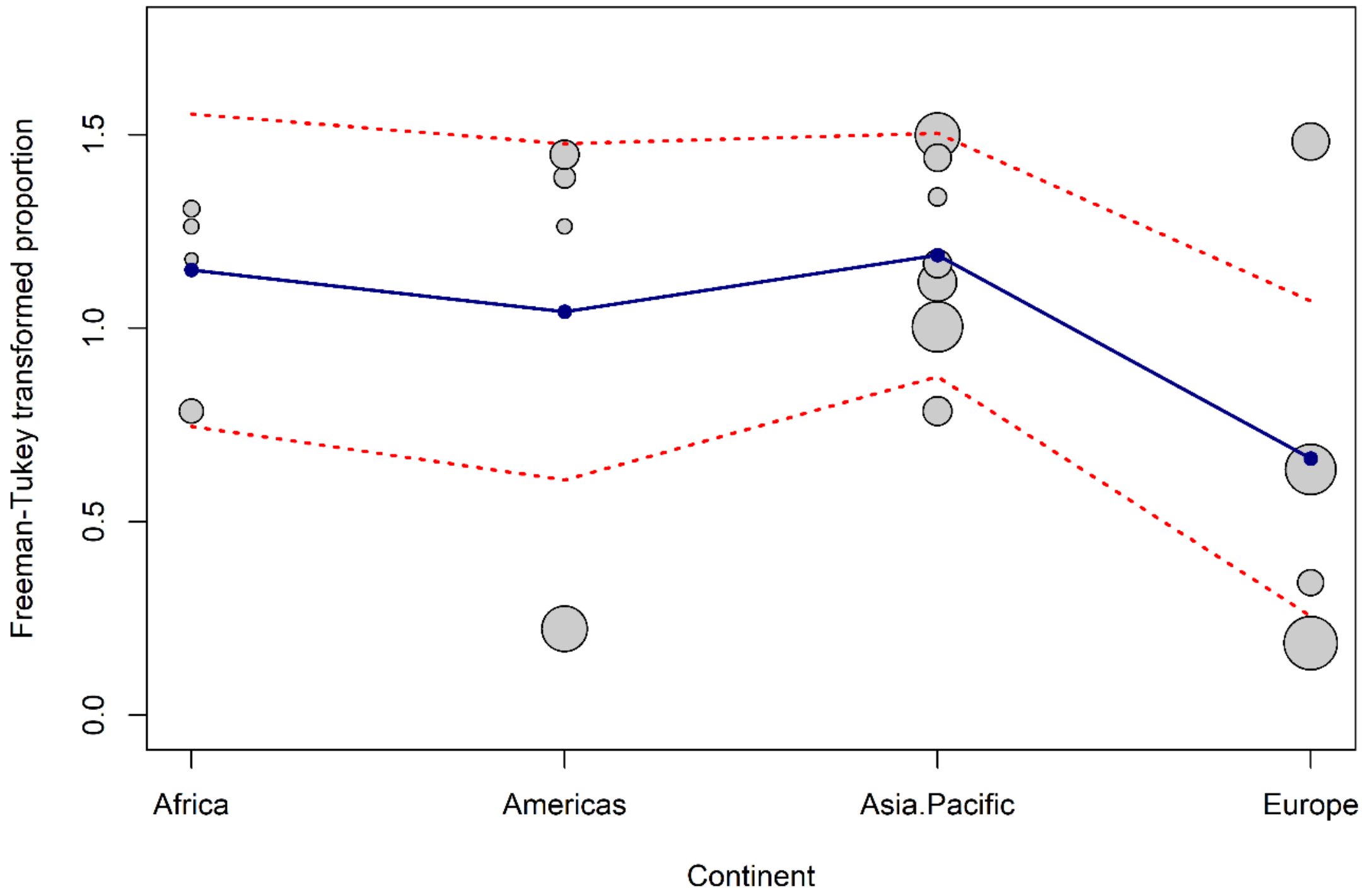

Figure S18. Univariate meta-regression of CRE-positive proportion based on publication year.

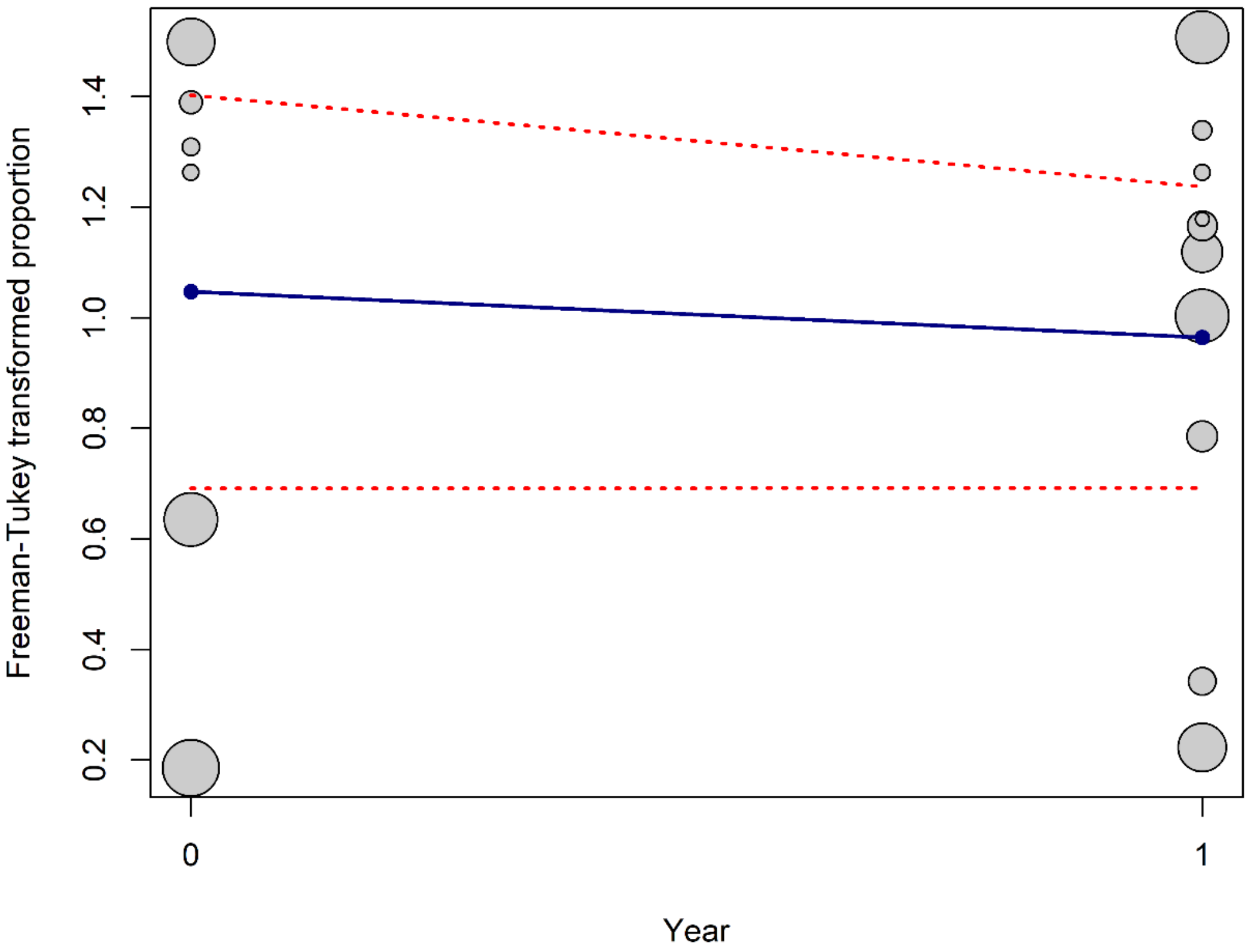

Figure S19. Univariate meta-regression of CRE-positive proportion based on sampling environment.

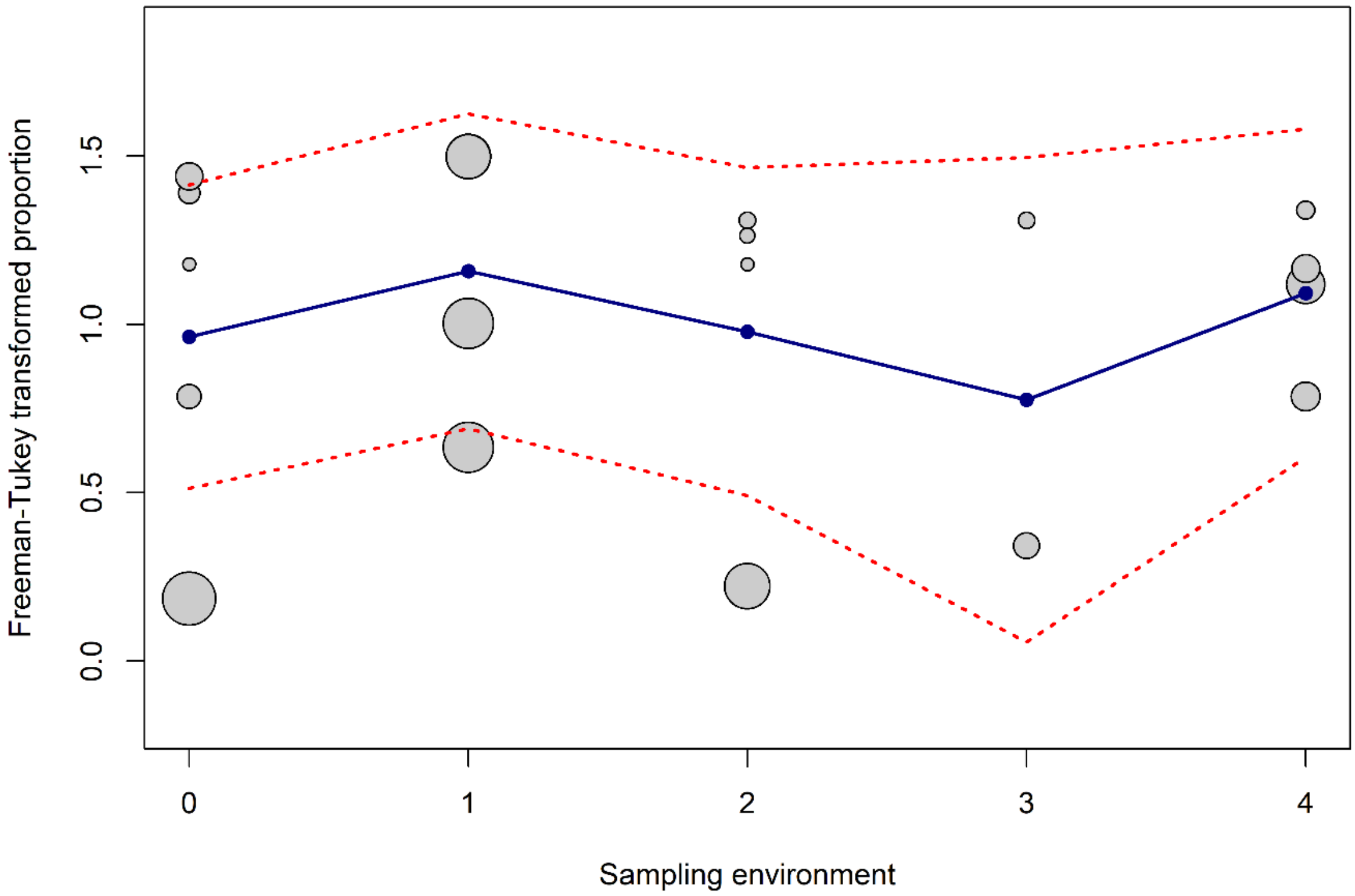

Supplement: Supplementary file 1 [file Data_Sheet_1.pdf]
